# Quantitative assessment of the vertebral pneumaticity in an anhanguerid pterosaur using micro-CT ﻿scanning

**DOI:** 10.1038/s41598-021-97856-6

**Published:** 2021-09-21

**Authors:** Richard Buchmann, Borja Holgado, Gabriela Sobral, Leonardo dos Santos Avilla, Taissa Rodrigues

**Affiliations:** 1 Laboratório de Paleontologia, Departamento de Ciências Biológicas, Centro de Ciências Humanas e Naturais, Universidade Federal do Espírito Santo, Vitória, ES 29075-910 Brazil; 2 Laboratório de Mastozoologia, Departamento de Zoologia, Universidade Federal do Estado do Rio de Janeiro, Rio de Janeiro, RJ 22290-240 Brazil; 3 Laboratório de Sistemática e Tafonomia de Vertebrados Fósseis, Departamento de Geologia e Paleontologia, Museu Nacional / Universidade Federal do Rio de Janeiro, Rio de Janeiro, RJ 20940-040 Brazil; 4 Research Group of Computational Paleobiology, Evolutionary Paleobiology Area, Institut Català de Paleontologia Miquel Crusafont, C/ de Les Columnes, Universitat Autònoma de Barcelona, 08193 Cerdanyola del Vallès, Catalonia Spain; 5 Staatliches Museum für Naturkunde, 70191 Stuttgart, Germany

**Keywords:** Palaeontology, Evolution, Zoology, Anatomy

## Abstract

Research on the postcranial skeletal pneumaticity in pterosaurs is common in the literature, but most studies present only qualitative assessments. When quantitative, they are done on isolated bones. Here, we estimate the Air Space Proportion (ASP) obtained from micro-CT scans of the sequence from the sixth cervical to the fourth dorsal vertebra of an anhanguerine pterosaur to understand how pneumaticity is distributed in these bones. Pneumatisation of the vertebrae varied between 68 and 72% of their total volume. The neural arch showed higher ASP in all vertebrae. Anhanguerine vertebral ASP was generally higher than in sauropod vertebrae but lower than in most extant birds. The ASP observed here is lower than that calculated for the appendicular skeleton of other anhanguerian pterosaurs, indicating the potential existence of variation between axial and appendicular pneumatisation. The results point to a pattern in the distribution of the air space, which shows an increase in the area occupied by the trabecular bone in the craniocaudal direction of the vertebral series and, in each vertebra, an increase of the thickness of the trabeculae in the zygapophyses. This indicates that the distribution of pneumatic diverticula in anhanguerine vertebrae may not be associated with stochastic patterns.

## Introduction

Bones possessing internal air diverticula are called pneumatic bones. They differ from the non-pneumatic ones in that they present lower vascularisation as well as pneumatic foramina on their surface, which are responsible for the entrance of air into the bone through air sacs^1^. Among extant vertebrates, postcranial skeletal pneumaticity is restricted to birds^2^, but it is widely present in extinct taxa, such as pterosaurs and several nonavian dinosaurs^3–11^, thus yielding different hypotheses on the evolutionary emergence of this feature in archosaurs^3,4,6–18^.

Previous quantitative studies that analysed sections of long bones of birds used the variable *K* to calculate the proportion of internal space in tubular bones^19,20^. In order to estimate this proportion in bones of other shapes, such as vertebrae and epiphyses of long bones, Wedel^21^ defined the Air Space Proportion (ASP): the proportion of the volume of a given bone—or the area of a bone section—filled by air. The method was developed using sauropodomorph vertebrae^21^.

**Figure 1:**
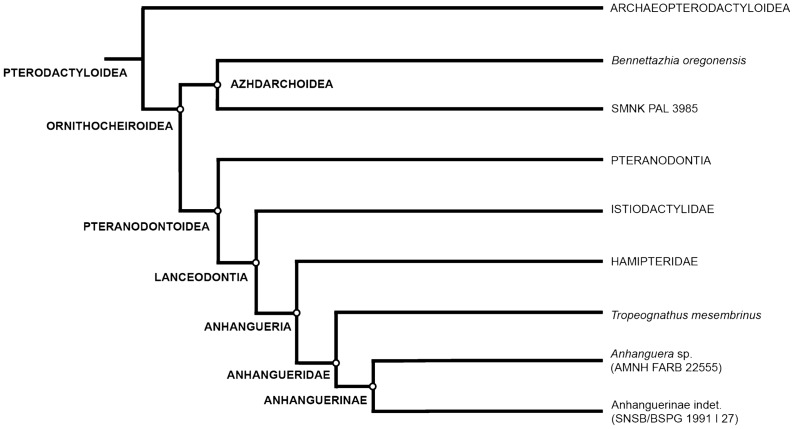
Simplified phylogenetic proposal of the Pterodactyloidea ingroup relationships displaying pterosaur specimens relevant in this work. General tree topology after Kellner^30^, Rodrigues and Kellner^31^, Holgado et al.^32^ and Holgado and Pêgas^33^.

More recently, the ASP method has been applied to pterosaurs, but in a limited way. Elgin and Hone^22^ calculated the ASP of the exposed cross-sections of some bones from the same individual, including an undetermined cervical vertebra, a rib, and a few appendicular elements. Martin and Palmer^23,24^ determined the ASP of long bones of different specimens using high-resolution X-ray computed tomography (or micro-CT) scans, allowing measurements along virtual sections. However, studies that analyse the degree of pneumaticity of the axial skeleton of pterosaurs more broadly are still lacking. Claessens et al.^15^ analysed a single vertebra (the sixth cervical) of the pterosaur specimen AMNH 22555 (referred to *Anhanguera*^25,26^ and stored at the American Museum of Natural History, New York, USA), but they did not calculate the ASP. These analyses have contributed to our understanding of pterosaur pneumaticity, but because they are restricted to sampling one vertebra or bone region, they are of limited use to understand how pneumaticity could vary within an individual, species, or at broader phylogenetic levels. The ASP is a reliable and well-established quantitative method that can be used to assess postcranial pneumaticity patterns more accurately and to explore the relationship between them and the evolutionary history or ecology of a group. Here, we explore such patterns of pneumaticity through micro-CT scans of the specimen stored at the Staatliche Naturwissenschaftliche Sammlungen Bayerns/Bayerische Staatssammlung für Paläontologie und Geologie, Munich, Germany, SNSB/BSPG 1991 I 27. The fossil comes from the Romualdo Formation of the Araripe Basin (Santana Group) and is Late Aptian (Early Cretaceous) in age^27,28^. It was described by Veldmeijer et al.^29^, who tentatively identified it as *Brasileodactylus* sp., but due to the lack of genus-level diagnostic features, here we restrict its identification to the Anhanguerinae.^29–33^ (Fig. 1).

**Figure 2:**
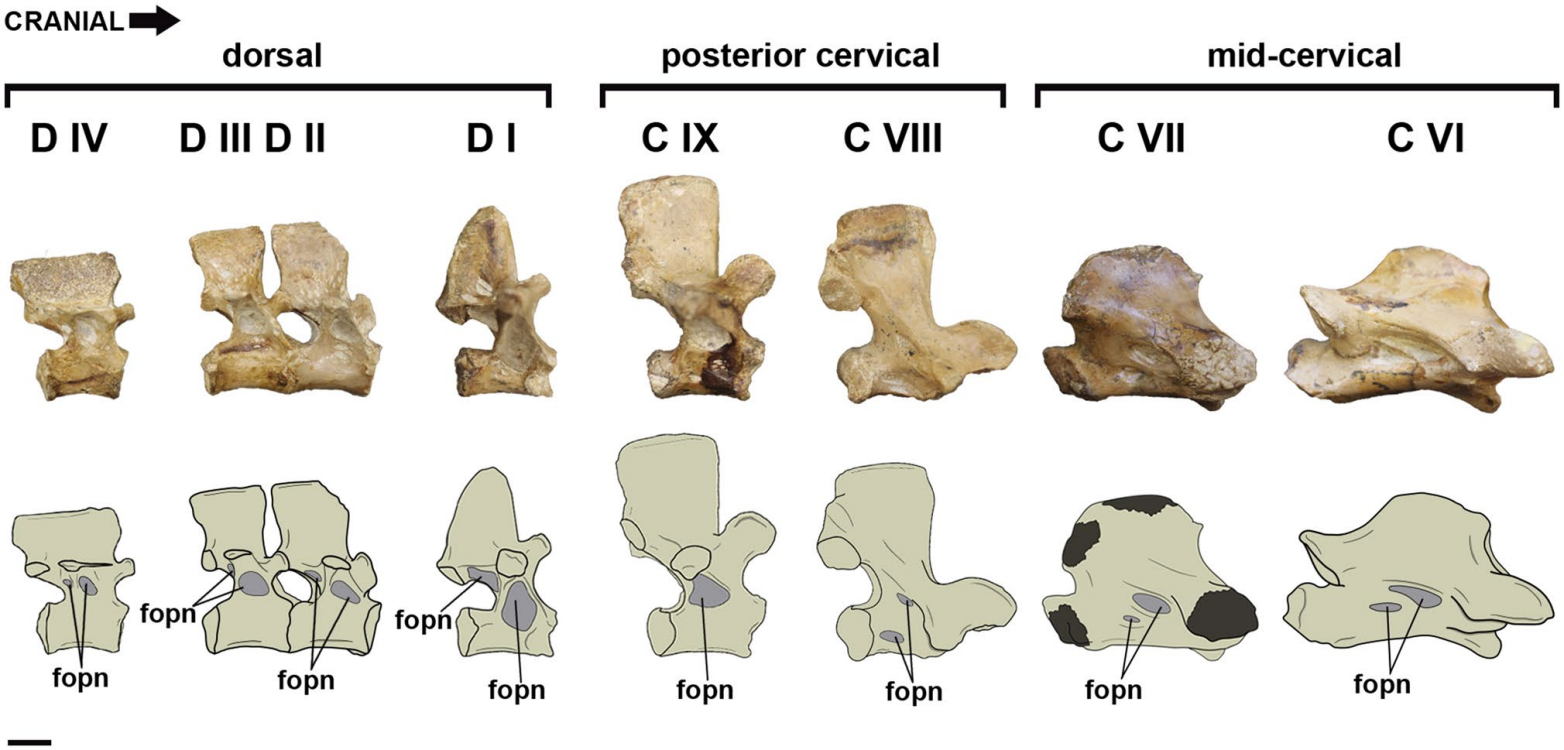
Scanned vertebral sequence belonging to SNSB/BSPG 1991 I 27. Abbreviations: fopn, pneumatic foramen. Scale bar: 10 mm.

**Figure 3:**
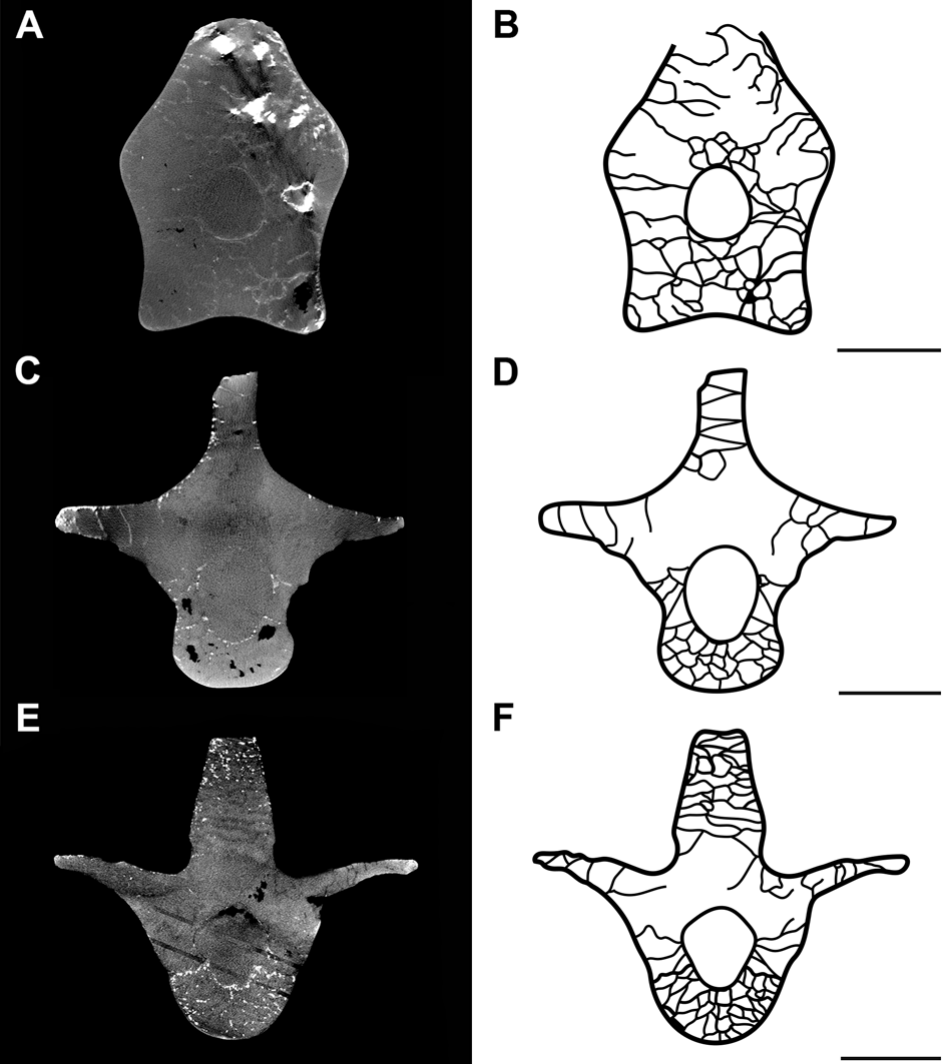
The internal architecture of trabeculae in the mid-length of the vertebrae**.** Slices of the scans and interpretative drawings of the mid-length of the seventh cervical vertebra (**A, B**) and second (**C, D**) and fourth (**E, F**) dorsal vertebrae of SNSB/BSPG 1991 I 27. Trabeculae and cortical bone are in black in the drawings. Scale bar: 10 mm.

The material comprises an incomplete and non-osteologically mature skeleton with both cranial and postcranial elements (Veldmeijer et al.^29^: figs. 3, 5–11). Although the specimen includes a sequence from the sixth cervical to the tenth dorsal vertebra (Veldmeijer et al.^29^: figs. 5–6), we micro-CT scanned only from the sixth cervical to the fourth dorsal vertebra (Fig. 2). The more caudal dorsal vertebrae have extremely reduced pneumatic foramina and we expected a higher variation in pneumatisation in the vertebral series near the base of the neck. The fossil has an excellent three-dimensional preservation, showing no significant signs of flattening, which allows the assessment of how pneumaticity varies along the vertebral series at the base of the neck. We calculated the ASP in consecutive bone sections of the vertebral series and suggest a more integrative approach for the issue. We aim to offer a more global understanding of the variation in pneumaticity patterns in the vertebral column of pterosaurs to provide a more accurate anatomical model for biomechanical studies.

## Results

### ASP values in the transverse sections

The transverse section of the mid-length of the neural arch was the most pneumatised section in all vertebrae analysed, with at least 77% of air space. In contrast, sections of the mid-length of the centra did not reach more than 73% of ASP, with the largest proportions observed in the seventh and ninth cervical vertebrae and in the first dorsal vertebra (Table 1).

**Table 1:** ASP values of the analysed transverse sections of the vertebrae of SNSB/BSPG 1991 I 27.

Vertebra/section	Cot	Cen	Con	Prz.r	Prz.l	Na	Poz.r	Poz.l
Cervical VI	0.73	0.71	0.75	0.65	0.68	0.78	–	0.74
Cervical VII	0.71	0.73	0.60	0.69	–	0.79	–	–
Cervical VIII	0.70	0.69	0.57	0.74	0.76	0.80	0.73	0.80
Cervical IX	0.56	0.73	0.70	0.62	0.80	0.84	0.72	0.69
Dorsal I	0.64	0.73	0.60	0.73	0.67	0.81	0.62	0.67
Dorsal II	0.70	0.63	0.57	0.69	0.75	0.77	0.69	0.66
Dorsal III	0.67	0.64	0.68	0.65	0.69	0.82	0.79	0.75
Dorsal IV	0.56	0.64	0.53	0.75	0.73	0.82	0.67	0.72

Cot, cotyle; Cen, mid-length of the centrum; Con, condyle; Prz.r, right prezygapophysis; Prz.l, left prezygapophysis; Na, mid-length of the neural arch; Poz.r, right postzygapophysis; Poz.l, left postzygapophysis. –, ASP not measured.

The mid-length section of the neural arches had higher ASP than the zygapophyses. However, the ASP of the mid-length of the centra is higher than the cotyle and condyle in the seventh and ninth cervical vertebrae, and in the first and fourth dorsal vertebrae. In the eighth cervical and the second dorsal vertebra, the ASP of the centra increase from the condyle to the cotyle (Table 1).

### Mean ASP values

Within the same vertebra, we found a higher mean ASP in the articulations of the neural arch (pre- and postzygapophyses) than in those of the centrum (cotyle and condyle), varying between 67 and 76% in the former and from 55 to 74% in the latter (Table 2). The sixth cervical vertebra was the only one that presented the opposite proportions.

**Table 2:** Mean ASP values of selected regions of the vertebrae of SNSB/BSPG 1991 I 27.

Vertebra/region	Cotyle and condyle	Zygapophyses	Centrum	Neural arch	Whole vertebra
Cervical VI	0.74	0.69	0.73	0.71	0.72
Cervical VII	0.66	0.69	0.68	0.74	0.70
Cervical VIII	0.64	0.76	0.65	0.77	0.72
Cervical IX	0.63	0.71	0.66	0.73	0.71
Dorsal I	0.62	0.67	0.66	0.70	0.68
Dorsal II	0.64	0.70	0.63	0.71	0.68
Dorsal III	0.68	0.72	0.66	0.74	0.71
Dorsal IV	0.55	0.72	0.58	0.74	0.68

ASP values did not vary significantly throughout the column, with vertebral means between 68 and 72%, with all analysed cervical vertebrae with at least 70% (Table 2). Except for the third, the dorsal vertebrae had slightly less pneumatisation than the cervical vertebrae.

Regarding the mean ASP of the centra, there was a general trend of decrease in pneumatisation from the cranial to the caudal vertebrae, with the highest mean ASP present in the sixth cervical and the lowest in the fourth dorsal (Table 2).

## Discussion

Although pterosaur vertebrae are generally significantly reduced in length compared to their limb bones, the extent of pneumaticity in the vertebrae of SNSB/BSPG 1991 I 27 varied between regions, whether closer to the articulation regions or in the mid-length of the vertebra, similar to what was observed in pterosaur long bones^23^. This indicates the importance of analysing ASP values in different sections of a given element, even when it is not particularly long.

The centrum has a laterally and dorso-ventrally compressed region in its mid-height and mid-length, respectively^34^. It is compact, with smaller air cavities and more trabeculae than the neural arch (Fig. 3). The increase in the proportion of trabecular bone in the centrum may be a biomechanical requirement to guarantee structural integrity and to confer more resistance to withstand the stresses caused by the movements naturally exerted by the base of the neck^35,36^, since the increase in trabeculae tends to increase elastic stability^37^.

**Figure 4:**
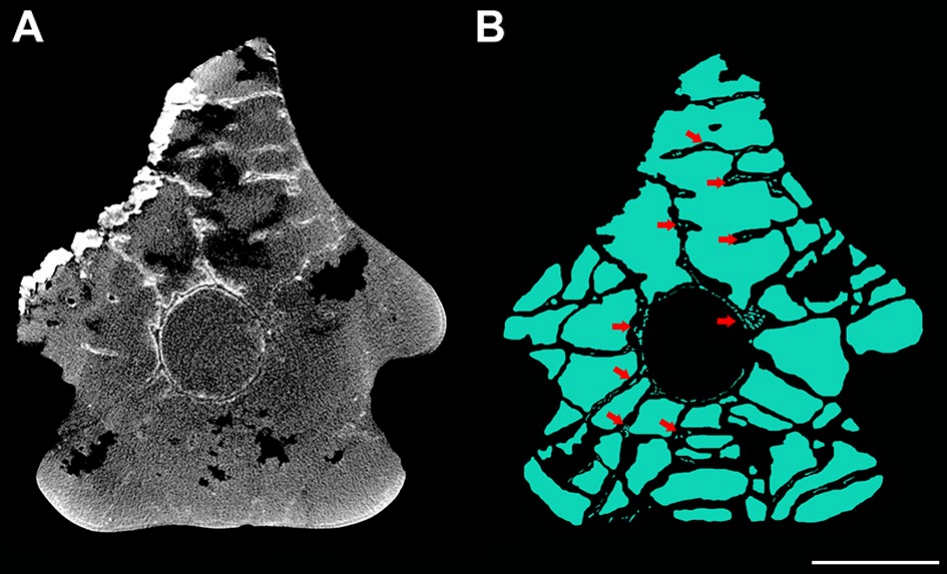
Internal air spaces in the mid-length of the vertebra. Slice of the scan (**A**) and internal air spaces marked in blue (**B**) in the mid-length of the vertebra of the sixth cervical vertebrae of SNSB/BSPG 1991 I 27. Red arrows indicate small hollow cavities within the bone trabeculae. Scale bar: 10 mm.

The mid-cervical vertebrae of SNSB/BSPG 1991 I 27 have no pneumatic foramina adjacent to the neural canal, only on the lateral surface of the centra^29^, differing from some other anhanguerians in which foramina are present in both regions^17,25,38^. Our analysis of the sections of SNSB/BSPG 1991 I 27 indicates that internal pneumatic cavities close to the sides of the centrum spread out dorsally and increase in the area of the pedicle of the neural arches, forming large air spaces above the neural canal and establishing a highly pneumatised region independent of the presence of pneumatic foramina in that region (Fig. 4).

The eighth and ninth cervical and the first dorsal vertebrae have pneumatic foramina on the bases of the transverse processes^29^, which are likely responsible for the entrance of air sinuses in the neural arches and in regions of the centra that are pneumatised. In these vertebrae, the centra are shorter than those of the mid-cervicals, a feature commonly observed in other ornithocheiroids^38–41^. Consequently, the air spaces of these centra are smaller than those of the mid-cervical vertebrae.

**Figure 5:**
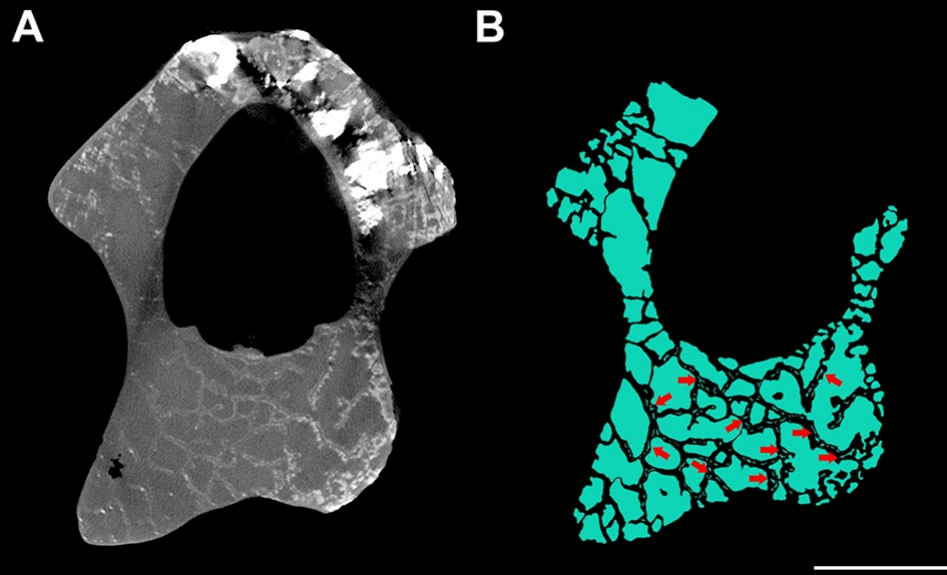
Internal air spaces in the condyle of the vertebra. Slice of the scan (**A**) and internal air spaces marked in blue (**B**) of the condyle of the seventh cervical vertebrae of SNSB/BSPG 1991 I 27. Red arrows indicate small hollow cavities within the bone trabeculae. Scale bar: 10 mm.

Most articular regions such as the zygapophyses, cotyle, and condyle presented less pneumatisation than the sections at the mid-length of the neural arches and the centra. This is expected as they need denser bone to increase resistance and absorb mechanical shocks^7,42–44^. However, differently than observed when comparing ASP of the neural arch and zygapophyses, the cotyle and condyle have pneumatisation at levels similar or higher than the mid-length section of the centrum (Table 1). This indicates that these structures may not follow a pneumatisation pattern similar to that observed in the centra. In addition, small hollow spaces within thicker bony trabeculae are commonly seen in the cotyle and condyle (Fig. 5), similar to those in the neural arch (Fig. 4). Although such cavities could actually be filled with air, they can also be associated with other soft tissues, such as blood vessels^45^.

The lower ASP values of the cotyle and condyle compared to those of the zygapophyses possibly indicate a more rigid articulation in the centrum. The sixth cervical is the exception, as it presented the most pneumatised cotyle and condyle of all analysed vertebrae (Table 2), suggesting that the support for resistance in the joints of the centrum of the middle cervical vertebrae probably require less of the presence of trabecular bone than in the posterior vertebrae^37^.

When considering the mean ASP of each vertebra of SNSB/BSPG 1991 I 27, the cervical vertebrae (range 0.70–0.72; median: 0.72) are slightly more pneumatised than the dorsal vertebrae (range 0.68–0.71; median: 0.68), while the mean ASP of their neural arches is equivalent (cervical vertebrae range 0.71–0.77; median: 0.735; dorsal vertebrae range 0.70–0.74; median: 0.725).

**Figure 6:**
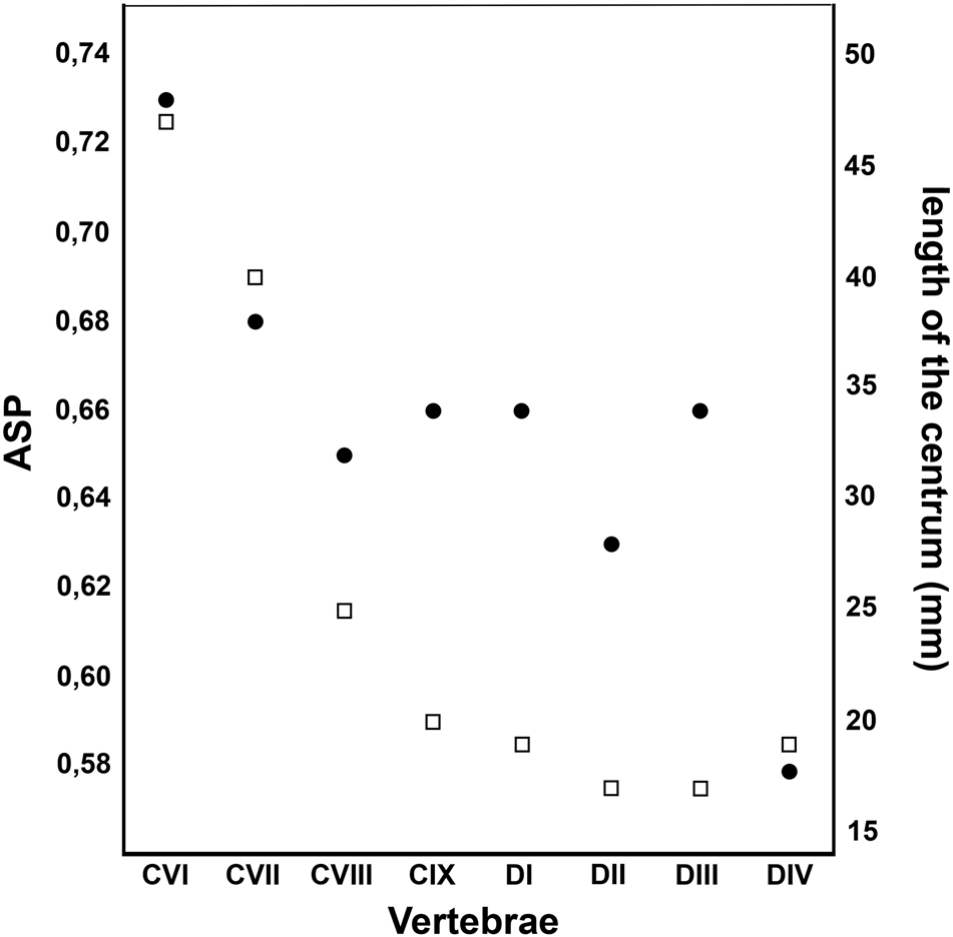
Relationship between pneumatisation and centrum length of the vertebrae. Mean vertebral ASP (black circles) and length of the centra (white squares) of the eight analysed vertebrae of SNSB/BSPG 1991 I 27. CVI–IX, sixth to ninth cervical vertebrae; DI–IV, first to fourth dorsal vertebrae. Mean ASP values from Table 2. Centrum length measurements from Veldmeijer et al.^29^ (2009).

However, a substantial decrease in air space is observed on the mean ASP of the centra between cervical and dorsal series (cervical vertebrae range 0.73–0.65; median 0.67; dorsal vertebrae range 0.66–0.58; median: 0.645), which may be related to the reduction of the length of their centra (Fig. 6). This decrease in ASP may also be a result of the increase in cortical and trabecular bone in this region of the vertebral column due to tensions caused by the movement at the base of the neck^36^, or indicate an additional need for other soft tissues in the centrum unrelated to the respiratory tract, as blood vessels^45^.

The increase or reduction of pneumatisation between different vertebrae—or between regions of a single vertebra—does not directly support inferences on the biomechanics of the neck of the pterosaur analysed here. To test our hypotheses, in the future we plan to carry out functional studies applying loads to pterosaur cervical vertebral series. In any case, the variation in pneumaticity presented here suggests a pattern of reduced pneumatisation in the cranio-caudal direction and a decrease between zygapophyses in relation to the neural arches. Therefore, we hypothesise that the quantitative disposition of the air space follows a pattern that may be related to the biomechanics of the neck^37^.The mean ASP per vertebra of SNSB/BSPG 1991 I 27 (range 0.68–0.72; median: 0.705) was lower than the value (0.83) presented for an indeterminate azhdarchoid cervical vertebra fragment stored at the Staatliches Museum für Naturkunde Karlsruhe, Karlsruhe, Germany, SMNK 3985^22^. However, the analysed cross-section seems to belong to the middle part of the neural arch, which data collected here shows to be more pneumatised than the centra. In this case, the ASP values of SNSB/BSPG 1991 I 27 and SMNK 3985 would be similar. Such high degree of pneumatisation had been already suggested for late pterodactyloids^17,18,22,46,47^.

The mean ASP of each vertebra analysed here was slightly lower than the mean ASP values of the six long bones of other anhanguerian specimens studied by Martin and Palmer^23^ (range 0.68–0.83; median: 0.76) (Table 3). A higher pneumatisation of the appendicular skeleton in relation to the axial is not unexpected, given the support function of the vertebral column, which tends to be more rigid. However, the azhdarchoid SMNK 3985 shows higher values in axial than in appendicular elements^22^, contrasting with anhanguerians. Nevertheless, the ASP of SMNK 3985 was estimated based only on one cross-section per element, which represent locations of particularly low density. Considering that pneumaticity varies throughout the length of the bones, as shown here, analyses in multiple cross-sections allow for more confident inferences on the ASP of a given element^23^.

**Table 3:** Mean ASP of the appendicular skeleton of eight pterodactyloid specimens (from Martin & Palmer23).

Specimen	Element	Measured ASP range	Mean ASP
NHMUK PV OR35228 (Anhangueria indet.)	Undetermined phalanx of digit IV	0.74–0.76	0.76
NHMUK PV OR39411 (Anhangueria indet.)	I phalanx of digit IV	0.68–0.88	0.77
NHMUK PV OR41637 (Anhangueria indet.)	I phalanx of digit IV	0.70–0.88	0.83
UP WP1 (Anhangueria indet.)	I phalanx of digit IV	0.77–0.87	0.81
UP WP2 (Anhangueria indet.)	II phalanx of digit IV	0.69–0.84	0.76
UP WP3 (Anhangueria indet.)	III phalanx of digit IV	0.59–0.76	0.68
USNM 11925 (*Bennettazhia oregonensis*)	Humerus	0.77–0.85	0.81

Martin & Palmer^23^ also determined the ASP of the humerus of the holotype of the tapejaromorph *Bennettazhia oregonensis* (Gilmore, 1928) (stored at the National Museum of Natural History, Washington, DC, USA, USNM 11925), which also has mean ASP higher than those observed in the vertebrae of SNSB/BSPG 1991 I 27. This seems to indicate that long bones are more pneumatised than the vertebrae in the Ornitocheiroidea, but a more thorough analysis on tapejaromorphs remains to be done.

While the vertebrae of SNSB/BSPG 1991 I 27 were more pneumatised at their mid-lengths, pterodactyloid long bones, in general, have higher ASP in the articular ends^23^. This is explained by the extremely thin trabeculae present at the epiphyses, which provide the expected resistance for this region and yet store a large proportion of air space^23^. This is the opposite of what is observed in the sections of the cotyle and condyle of the vertebrae of SNSB/BSPG 1991 I 27. Additionally, long bones have a thinner cortex at the epiphyses, contributing to reduce volume at the articular ends^23^. Since the biological significance of pneumatisation in axial and appendicular bones is different^18^, such differences in the distribution of pneumaticity between the two are expected. In the vertebrae, on the other hand, distribution of ASP seem to indicate that stress loads are higher in the articulation areas, and, therefore, denser bone would be needed to support them.

The cervical vertebrae of SNSB/BSPG 1991 I 27 are comparatively more pneumatised than vertebrae of the sauropod dinosaurs *Apatosaurus* sp. (specimen stored at the Oklahoma Museum of Natural History, Norman, Oklahoma, USA, OMNH 01094), *Brachiosaurus* sp. (specimen stored at the Earth Sciences Museum, Brigham Young University, Provo, Utah, USA, BYU 12866) and *Camarasaurus* sp. (OMNH 01313), which show no ASP higher than 60% (see Table 2). However, *Sauroposeidon proteles* (OMNH 53062) has comparatively higher ASP, as seen in the middle length and zygapophysis sections of the sixth cervical vertebra, indicating that at least some sauropods reached higher levels of vertebral pneumatisation (Table 4 – from Wedel^21^).

**Table 4:** ASP of three unidentified cervicals of the sauropod dinosaurs *Apatosaurus* sp., *Brachiosaurus* sp., and *Camarasaurus* sp., and of the sixth cervical vertebra of *Sauroposeidon proteles* (from Wedel^21^).

Specimen/section	Cot	Cen	Con	Prz	Poz	mean ASP
OMNH 01094 (*Apatosaurus* sp.)	0.32	0.52	0.69	–	–	0.51
BYU 12866 (*Brachiosaurus* sp.)	0.39	0.67	0.73	–	–	0.60
OMNH 01313 (*Camarasaurus* sp.)	0.50	0.52	0.49	–	–	0.50
OMNH 53062 (*Sauroposeidon proteles*)	–	0.74	–	0.89	0.75	0.79

Cot, cotyle; Cen, mid-length of the centrum; Con, condyle; Prz, prezygapophysis; Poz, postzygapophysis.

In *Brachiosaurus* sp. (BYU 12866) and *Apatosaurus* sp. (OMNH 01094), the condyles have significantly higher ASP than the cotyles (Table 4). This is also the case of the ninth cervical of SNSB/BSPG 1991 I 27, but opposite to the seventh and eighth cervical vertebrae (Table 1). On the other hand, the cotyle of these sauropods had very low pneumatisation, fewer than 40% ASP^21^, while the ASP in the least pneumatised articular end of any SNSB/BSPG 1991 I 27 cervical vertebrae was 56%.

Both *Brachiosaurus* and *Apatosaurus* presented higher ASP values in the condyle than in the cotyle, thus hinting to the possibility that pneumatisation in these species could increase gradually from one end to the other of the centrum. Analyses of vertebral series of sauropods are needed to test this hypothesis. Our results, however, do not indicate such pattern of pneumatisation along the vertebral centrum in the analysed anhanguerine pterosaur.

In comparison to extant birds, the vertebral ASP of SNSB/BSPG 1991 I 27 is slightly lower than those of the posterior cervical vertebrae of extant storks (Ciconiidae), but higher than the ASP of the vertebrae of their first and second neck segments^48^. However, the ASP in birds was not estimated by cross-sections, but rather measured from the vertebral total volume^48^. Unlike what we calculated for SNSB/BSPG 1991 I 27, the pneumaticity of the cervical vertebrae in storks increases posteriorly. These results suggest that the increase in pneumaticity may also be related to regions in which there is a reduction in the range of movement in some axes, and, consequently, the tensions that could exceed the limit of bone resistance also decrease^48–50^, contradicting previous hypotheses of bone reinforcement in this region of the vertebral column^36^. In the case of the vertebrae of SNSB/BSPG 1991 I 27, the higher degree of pneumaticity present in the mid-cervical vertebrae could be a reflection of their long length, resulting in a low range of movement and, consequently, a decrease of the tensions on this region of the neck. However, the absence of more cranial vertebrae in the specimen analysed here makes this inference impossible to be tested at the moment.

## Conclusions

The mean ASP for each vertebra of SNSB/BSPG 1991 I 27 varied between 68 and 72%. Furthermore, we observed here a reduction of the ASP and increase in the area occupied by trabecular bone in the cranio-caudal direction in the vertebral series of SNSB/BSPG 1991 I 27, which may be related to a biomechanical requirement of the vertebral column^36,37,48–50^. Within the same vertebra, ASP values in the neural arch were higher at mid-length and decreased towards the zygapophyses, indicating a probable need for a higher level of stiffness^44^. These results support the hypothesis that pneumatisation of vertebrae follows a quantitative pattern within each vertebra and along the vertebral column that is probably determined by hitherto unrecognised variables rather than a stochastic pattern in the distribution of pneumatic diverticula.

The vertebrae investigated here are less pneumatised than anhanguerian appendicular bones analysed previously, which might be explained due to the axial skeleton's structural support function. However, studies of the same individual should be performed for more robust inferences.

The cervical vertebrae of SNSB/BSPG 1991 I 27 are more pneumatised than most sauropod vertebrae so far examined, except for *Sauroposeidon proteles*. The increase in pneumatisation in the mid-cervical vertebrae of SNSB/BSPG 1991 I 27 also differs from the distribution of the pneumatisation observed in the vertebrae of extant storks. Considering the influence of biomechanics on the pneumatisation of bones, this may indicate differences in the tensions exerted on the cervical series between both archosaur groups. Quantitative assessments of bone pneumaticity have the potential to fill in the gaps in our knowledge on the evolution of postcranial pneumatisation in archosaurs.

## Methods

### CT scans and preparation of the slices

The CT scans were performed by GS at the Museum für Naturkunde Berlin, Germany, using an X-ray micro-CT Phoenix|X-ray Nanotom scanner by GE Healthcare. Scans of the sixth cervical were made with a 0.1 mm Cu filter, but the remaining ones had none. Each scan comprised 1440 slices. The software datos|x—acquisition version 1.5.3.1 was used to acquire the data and datos|x—reconstruction version 1.5.0.22—64 bit to reconstruct the images in a three-dimensional file. Settings for different scans are listed in Table 5.

**Table 5:** Voltage, current and voxel size of each scanned element.

Vertebra/settings	Voltage (kV)	Current (mA)	Exposure (ms)	Voxel size (mm)
Cervical VI	100	350	1000	27.31
Cervical VII	95	300	1000	24.24
Cervical VIII	95	350	750	22.88
Cervical IX	100	250	750	23.66
Dorsal I	100	250	750	23.66
Dorsal II and III	100	300	750	20.97
Dorsal IV	120	210	500	17.34

The images were exported as DICOM files with the software Volume Graphics to visualise the individual slices. The grey balance of each image was enhanced using ImageJ^48^ to observe the pneumatic cavities, for such the same brightness/contrast value was used for all analysed slices.

### Air Space Proportion

**Figure 7:**
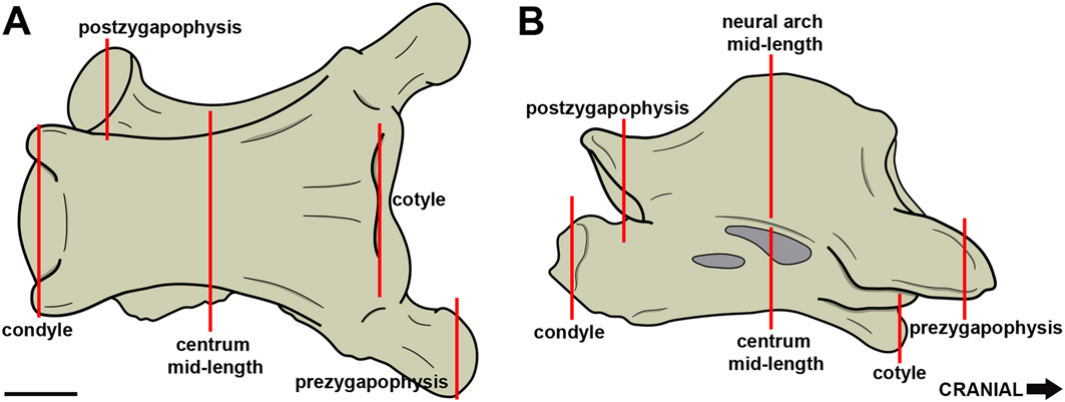
Transverse sections used for ASP calculation. Light grey: pneumatic foramina. Scale bar: 10 mm.

The identification of trabecular bone in some regions of the medullary space of the vertebrae on CT scans requires extreme caution. We excluded regions with poor contrast that were difficult to visualize from our analysis and selected cross-sections that showed no beam hardening or other obfuscating effects. The Air Space Proportion (ASP)^21^ was calculated to compare the air volume within each vertebral region. The ASP is the ratio of air space to the total area (cortical bone + medullary space) of a transversal section, with results varying from 0 to 1, with a larger value indicating higher bone pneumatisation^11,21^. Since the values are obtained from a single cross-section, they will not be representative of the whole structure^21^. We chose the following transverse sections on each vertebra for assessment (Fig. 7): 1. vertebral centrum at mid-length; 2. neural arch at mid-length; 3. cotyle; 4. condyle; 5. prezygapophysis; and 6. postzygapophysis. Regions that are likely to require more bone stiffness and elasticity in the vertebrae^43^ were considered in order to analyse how pneumatisation is distributed within each vertebral region. When these sections were totally or partially absent or damaged, the measurement was not taken. All cross-sections used are available as figures in the supplementary information (Supplementary Fig. S1–S35) and at 10.6084/m9.figshare.15152331.

We used Photoshop CS6 to recognise and segment the areas of the internal cavities, based on their differences in colouration (= density) to the bones. Using ImageJ software^51^, we converted the scale from millimetres to pixels and the areas were then measured in pixels. The obtained values were used to calculate the ASP of each transverse section. All total values for the cross-sections and areas identified as air cavities are available in Supplementary Table S1. Mean ASP values of selected regions of each vertebra were also determined: the cotyle and condyle taken together, all zygapophyses (left and right pre- and postzygapophysis), centrum (cotyle, condyle, and mid-length sections), neural arch (all measured zygapophyses and the mid-length section), and whole vertebra (calculated from all measured sections). The ASP was then compared between different sections, regions, vertebrae, and with those of sauropod vertebrae and the bones of pterosaurs already described in the literature.

## Supplementary Information

Supplementary Information.

## Data Availability

The published article includes all the data generated in the text. The analysed slices, the total areas of the air cavities and the total volume of each cross-section of the vertebrae can be found in the supplementary information. The slices are also available in Figshare (10.6084/m9.figshare.15152331).
