# Supplementary material for: Quantitative assessment of the vertebral pneumaticity in an anhanguerid pterosaur using micro-CT ﻿scanning
Source: Sci Rep. 2021 Sep 21;11:18718. doi: 10.1038/s41598-021-97856-6 (PMC8455612; doi:10.1038/s41598-021-97856-6)
Supplement: Supplementary file 1 — Supplementary Information. [file 41598_2021_97856_MOESM1_ESM.pdf]

Supplemental Information

QUANTITATIVE ASSESSMENT OF THE VERTEBRAL PNEUMATICITY OF A PTEROSAUR USING MICRO-CT SCANNING

Richard Buchmann, Borja Holgado, Gabriela Sobral, Leonardo dos Santos Avilla and Taissa Rodrigues

Supplementary Figures
Cross sections used for the analysis of the Air space proportion

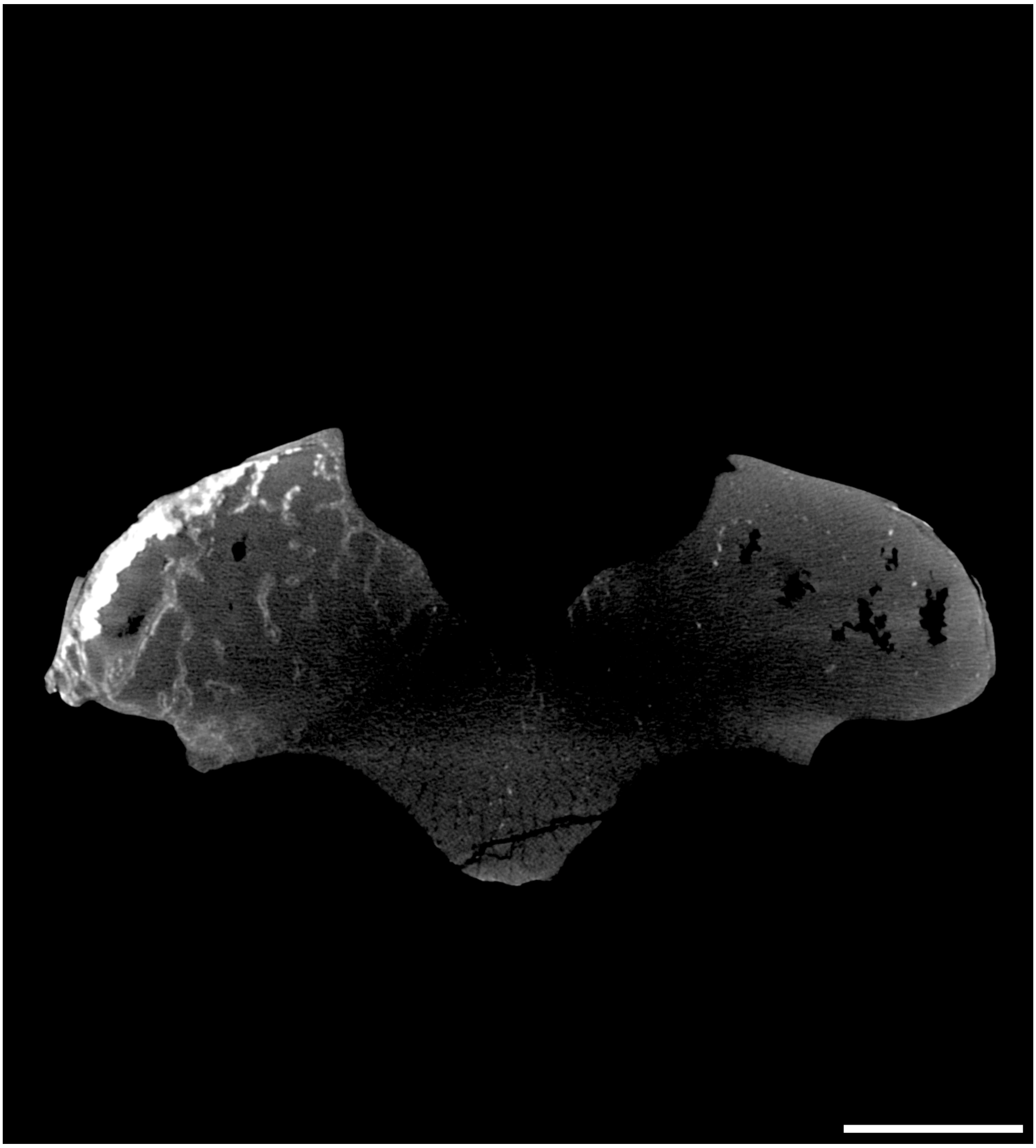

Supplementary Figure S1. Cross section of the cotyle of the sixth cervical vertebra belongs to SNSB/BSPG 1991 I 27. Scale bar: 10 mm.

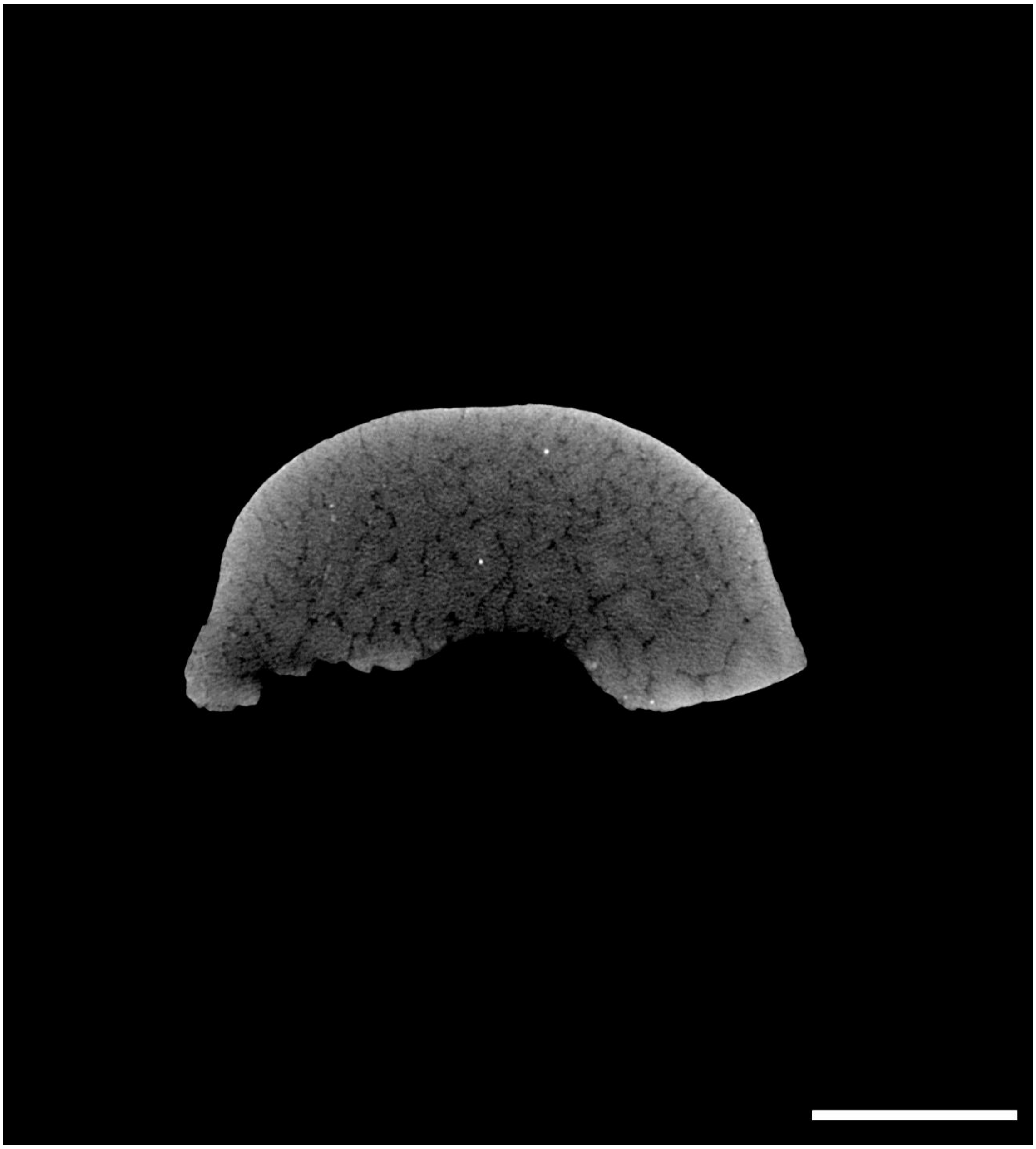

Supplementary Figure S2. Cross section of the condyle of the sixth cervical vertebra belongs to SNSB/BSPG 1991 I 27. Scale bar: 10 mm.

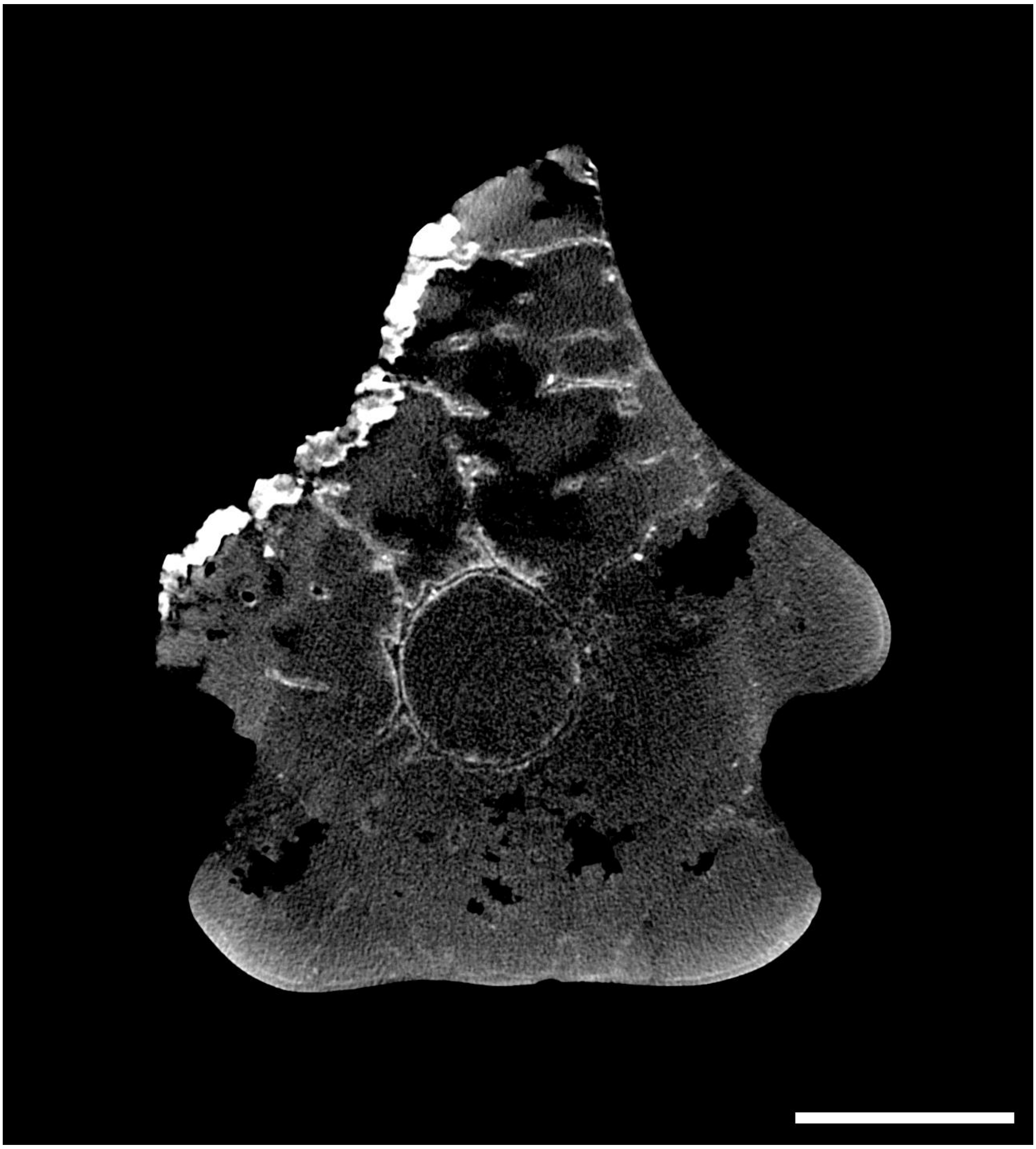

Supplementary Figure S3. Cross section of the mid-length of the sixth cervical vertebra belongs to SNSB/BSPG 1991 I 27. Scale bar: 10 mm.

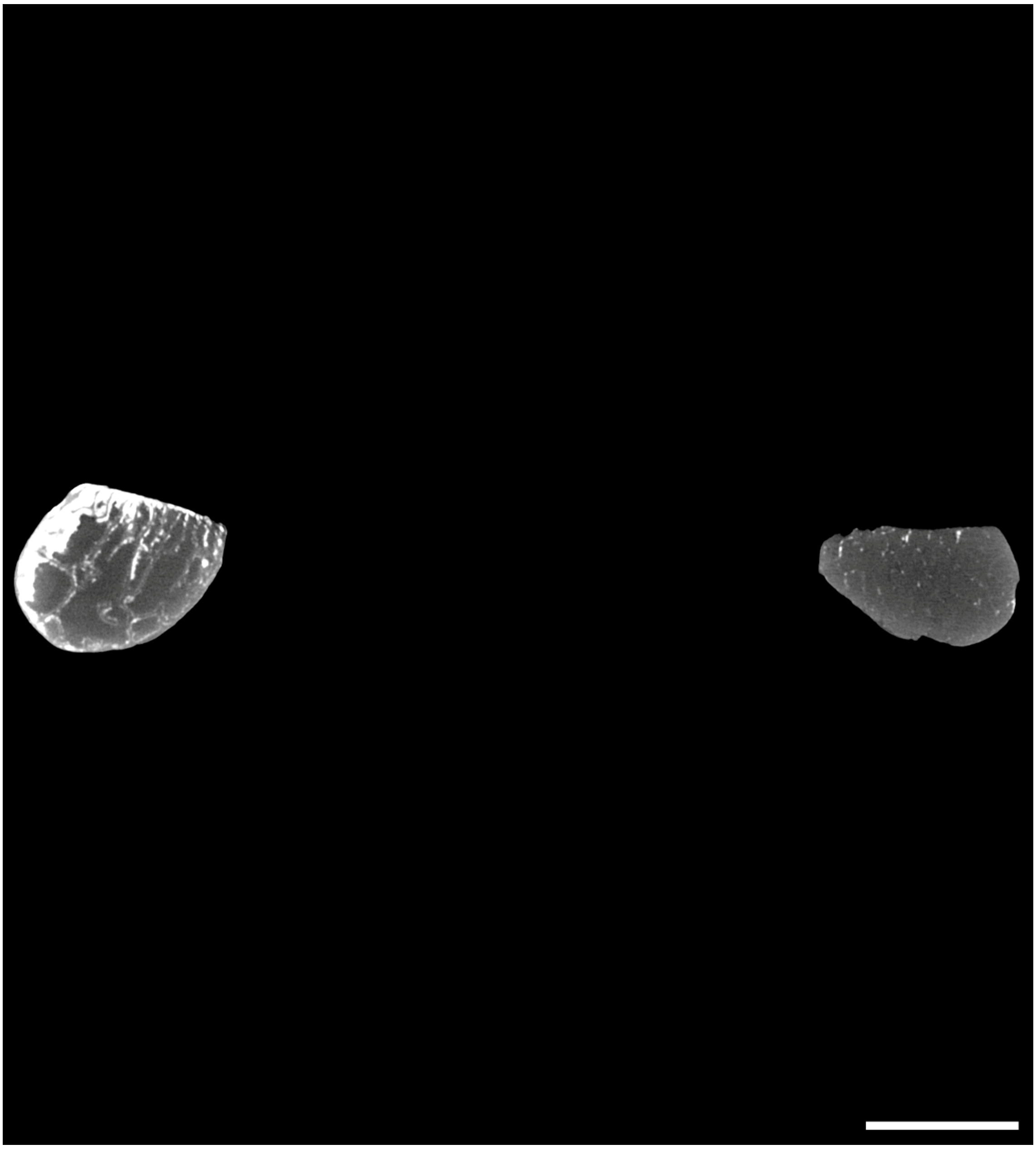

Supplementary Figure S4. Cross section of the prezygapophyses of the sixth cervical vertebra belongs to SNSB/BSPG 1991 I 27. Scale bar: 10 mm.

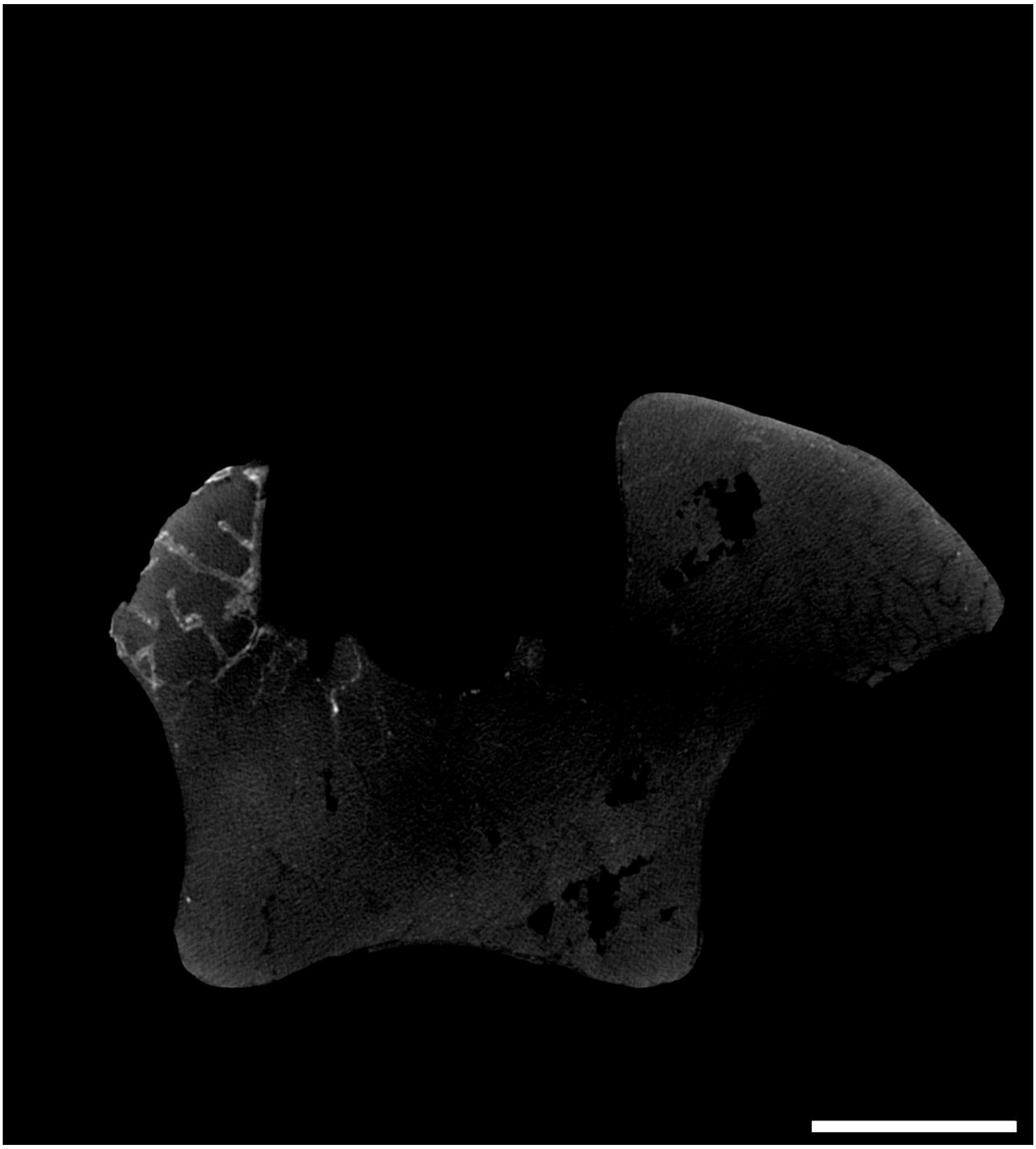

Supplementary Figure S5. Cross section of the left postzygapophysis of the sixth cervical vertebra belongs to SNSB/BSPG 1991 I 27. Scale bar: 10 mm.

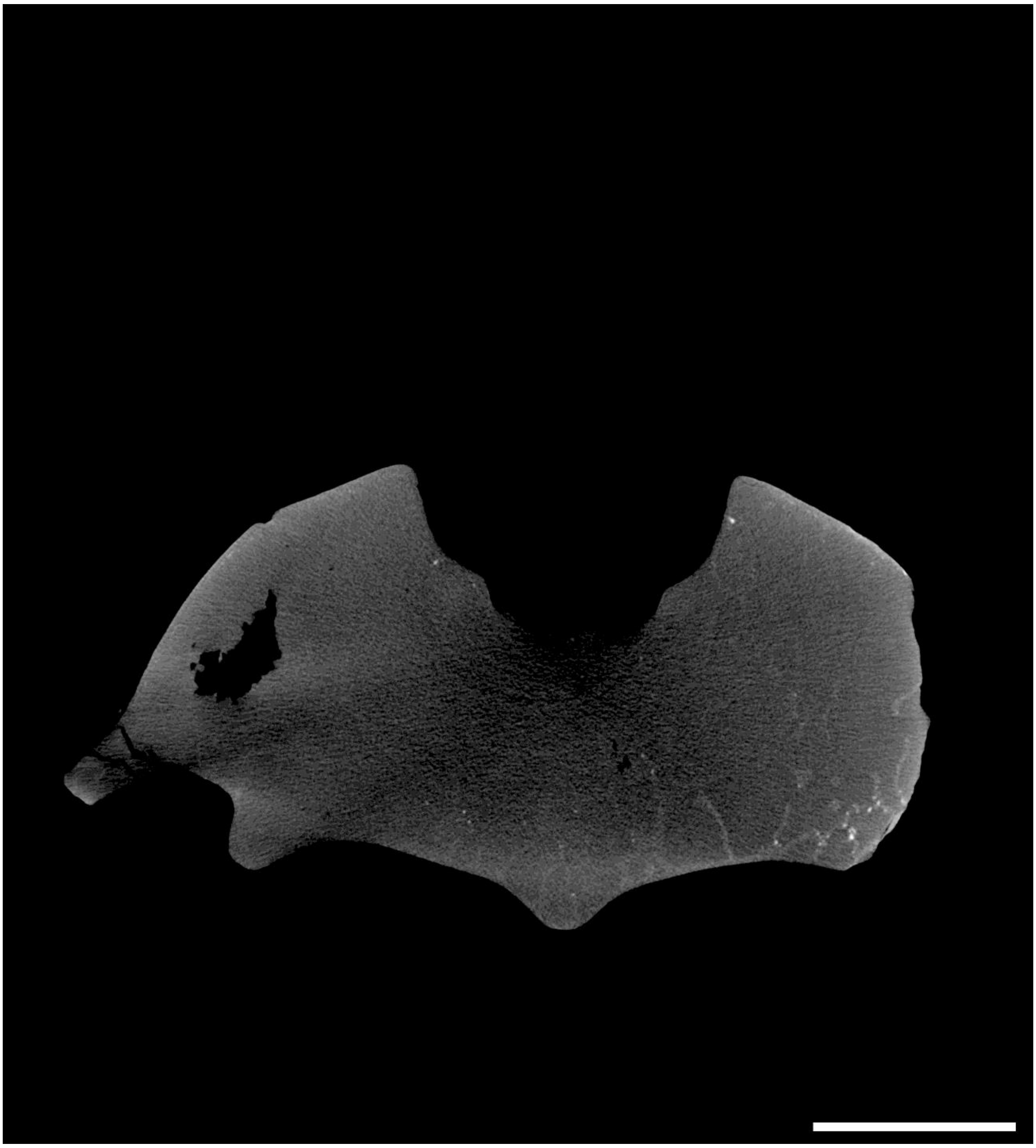

Supplementary Figure S6. Cross section of the cotyle of the seventh cervical vertebra belongs to SNSB/BSPG 1991 I 27. Scale bar: 10 mm.

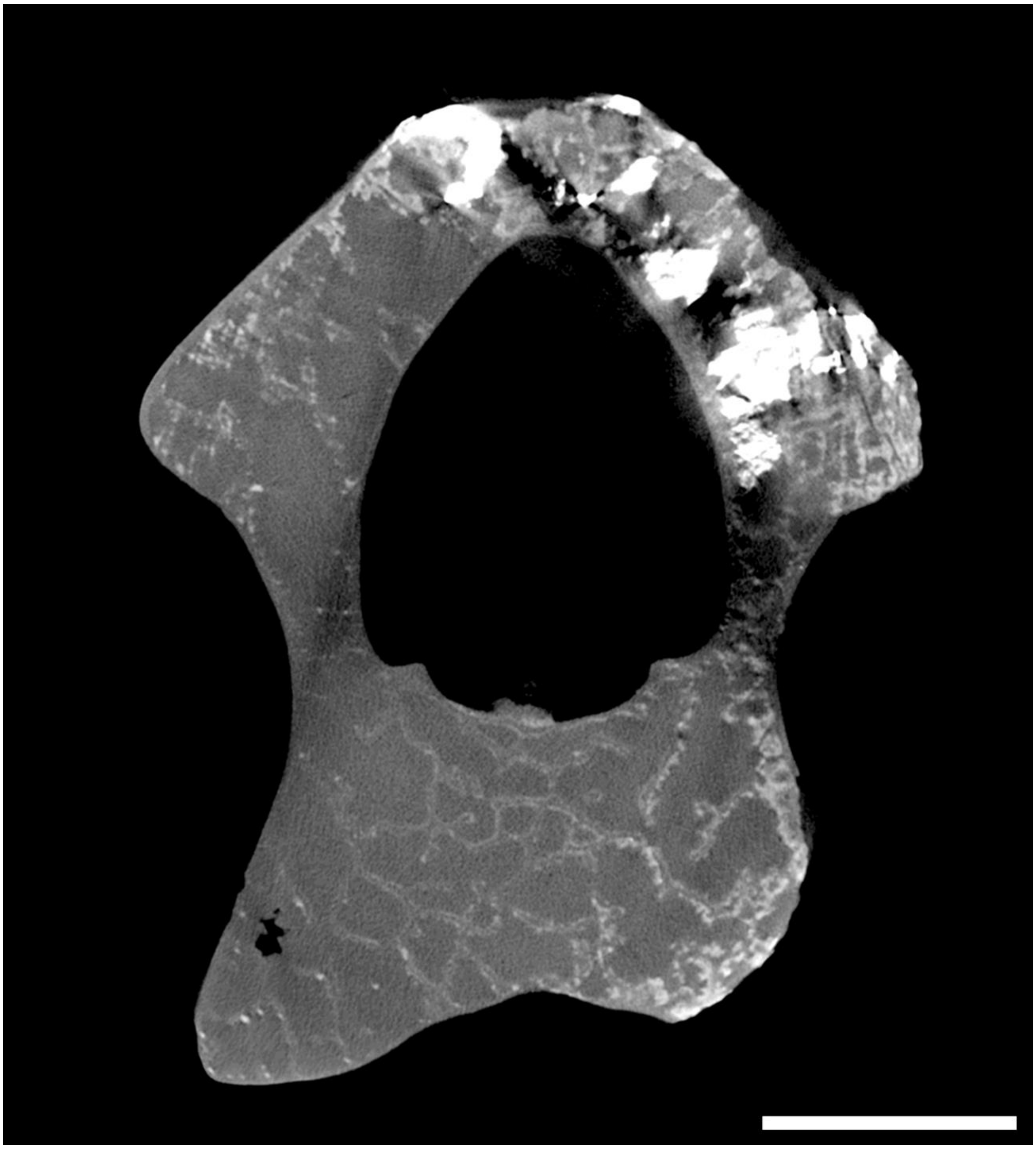

Supplementary Figure S7. Cross section of the condyle of the seventh cervical vertebra belongs to SNSB/BSPG 1991 I 27. Scale bar: 10 mm.

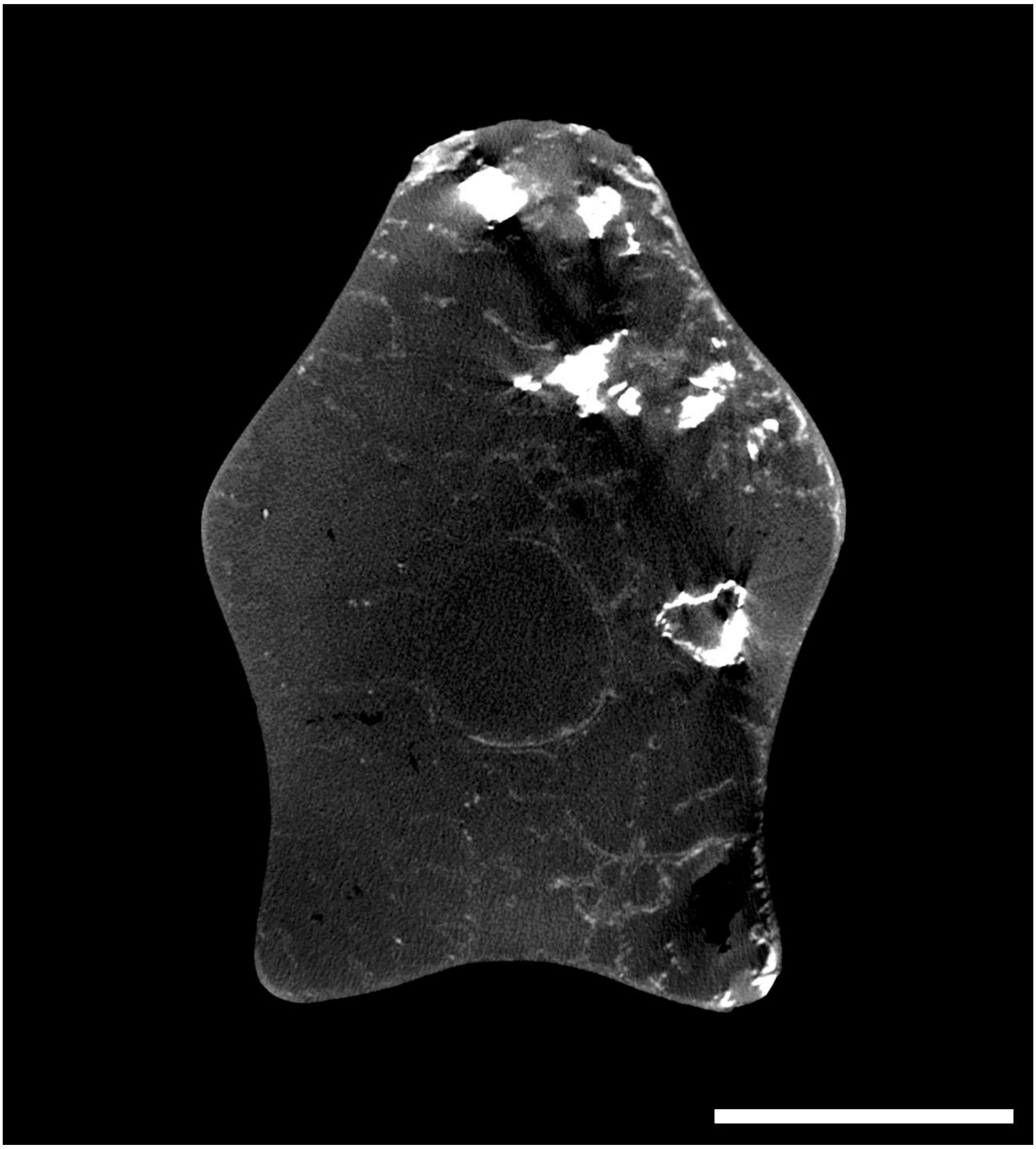

Supplementary Figure S8. Cross section of the mid-length of the seventh cervical vertebra belongs to SNSB/BSPG 1991 I 27. Scale bar: 10 mm.

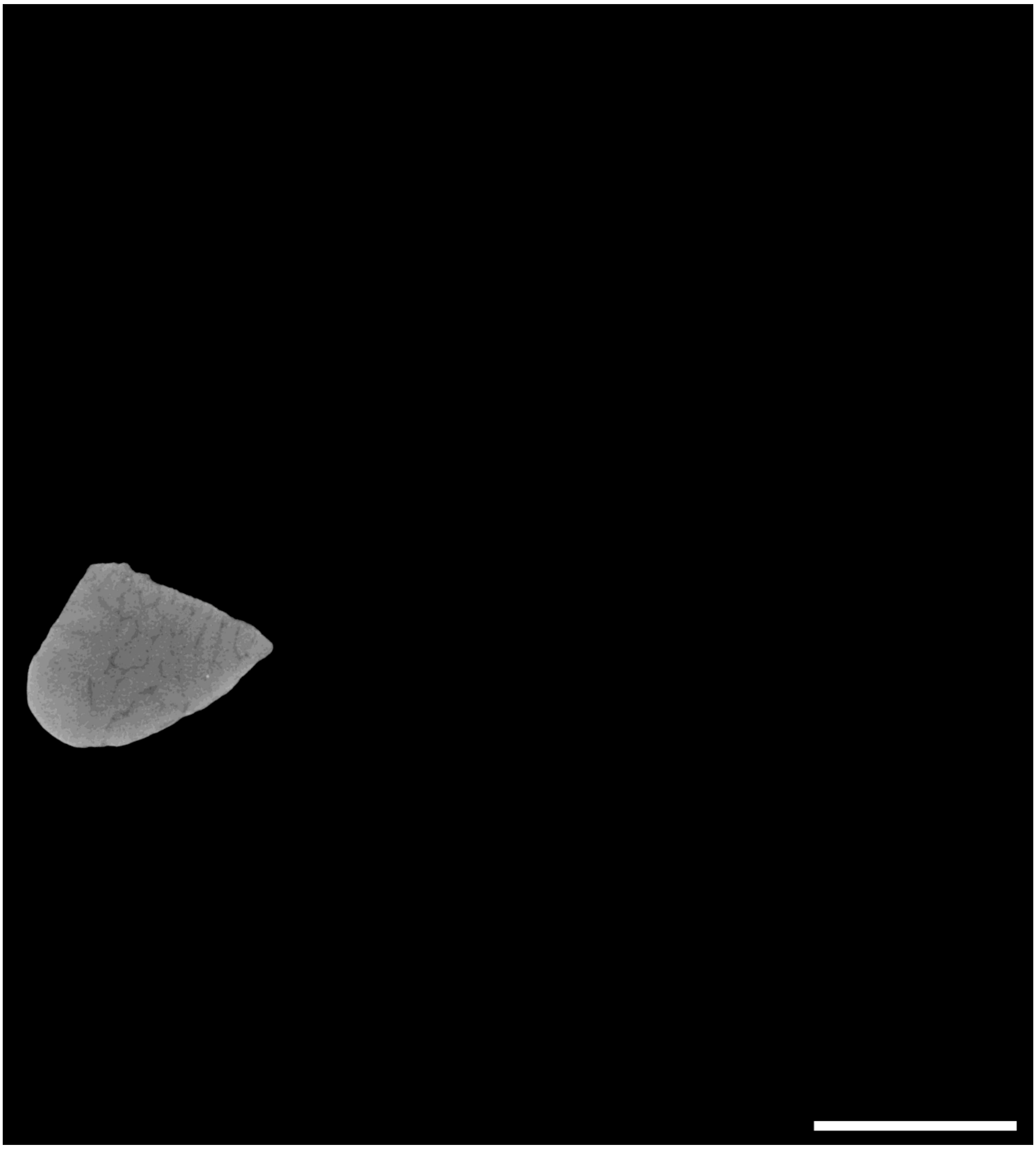

Supplementary Figure S9. Cross section of the right prezygapophysis of the seventh cervical vertebra belongs to SNSB/BSPG 1991 I 27. Scale bar: 10 mm.

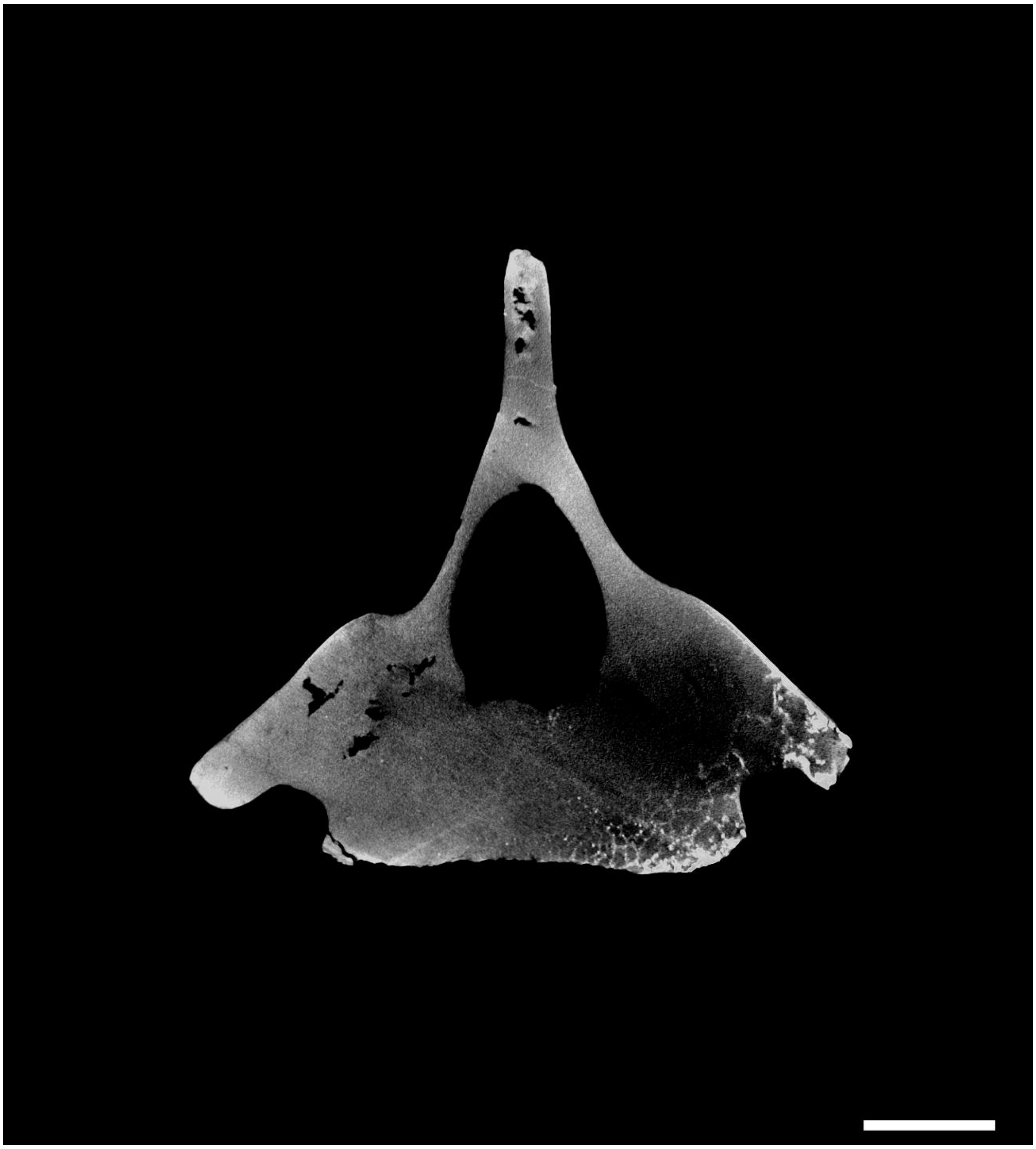

Supplementary Figure S10. Cross section of the cotyle of the eighth cervical vertebra belongs to SNSB/BSPG 1991 I 27. Scale bar: 8 mm.

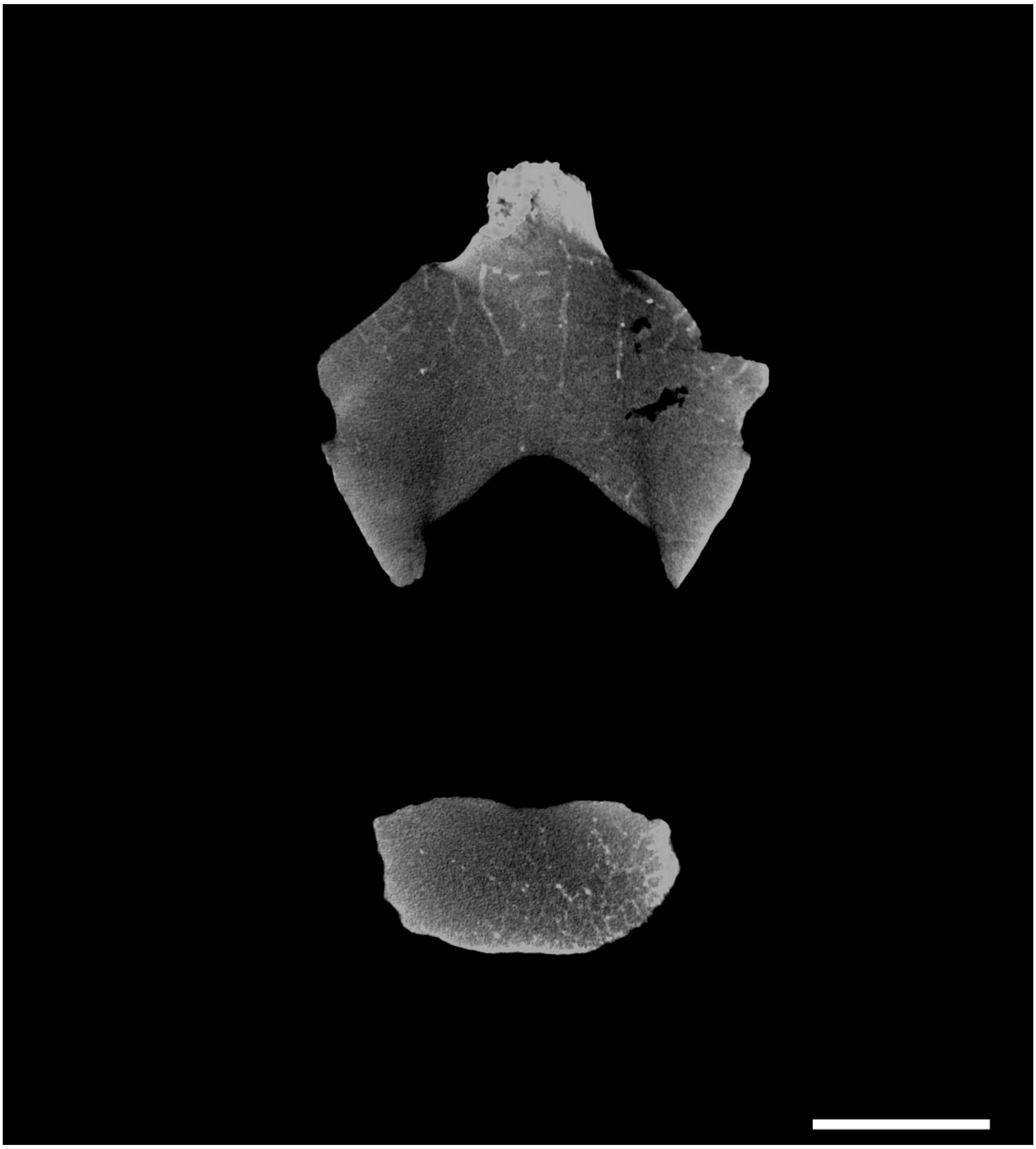

Supplementary Figure S11. Cross section of the condyle of the eighth cervical vertebra belongs to SNSB/BSPG 1991 I 27. Scale bar: 8 mm.

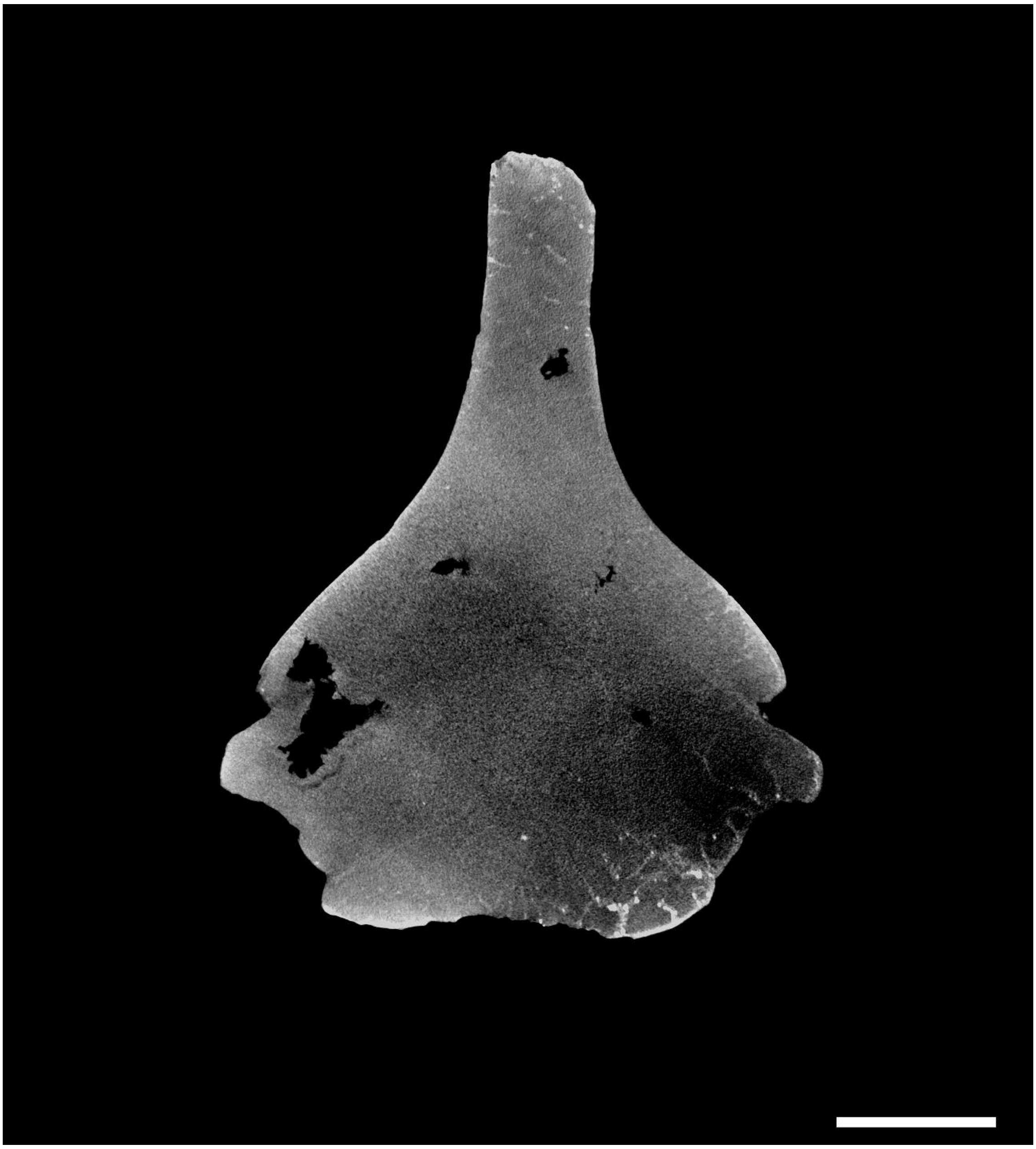

Supplementary Figure S12. Cross section of the mid-length of the eighth cervical vertebra belongs to SNSB/BSPG 1991 I 27. Scale bar: 8 mm.

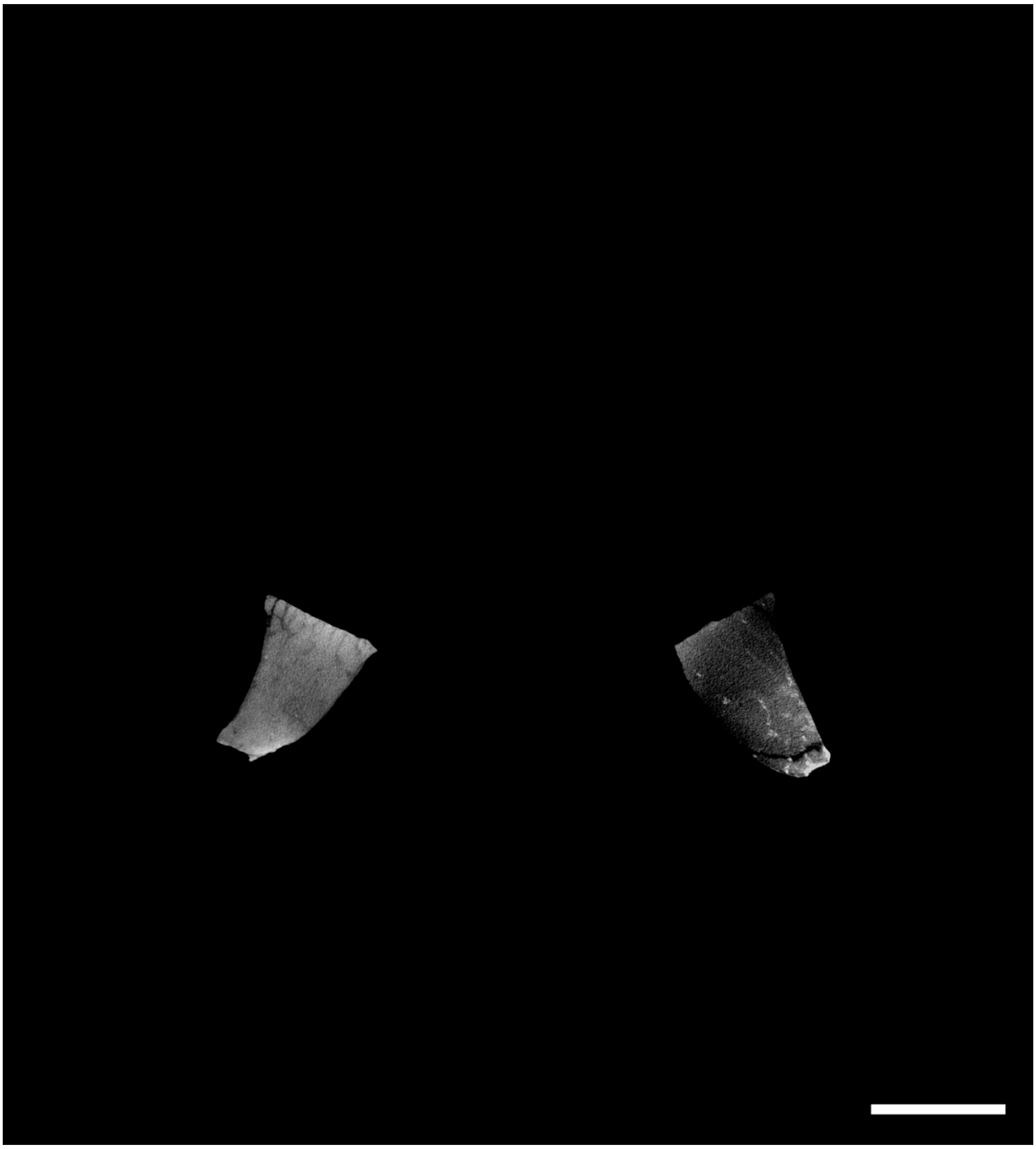

Supplementary Figure S13. Cross section of the prezygapophyses of the eighth cervical vertebra belongs to SNSB/BSPG 1991 I 27. Scale bar: 8 mm.

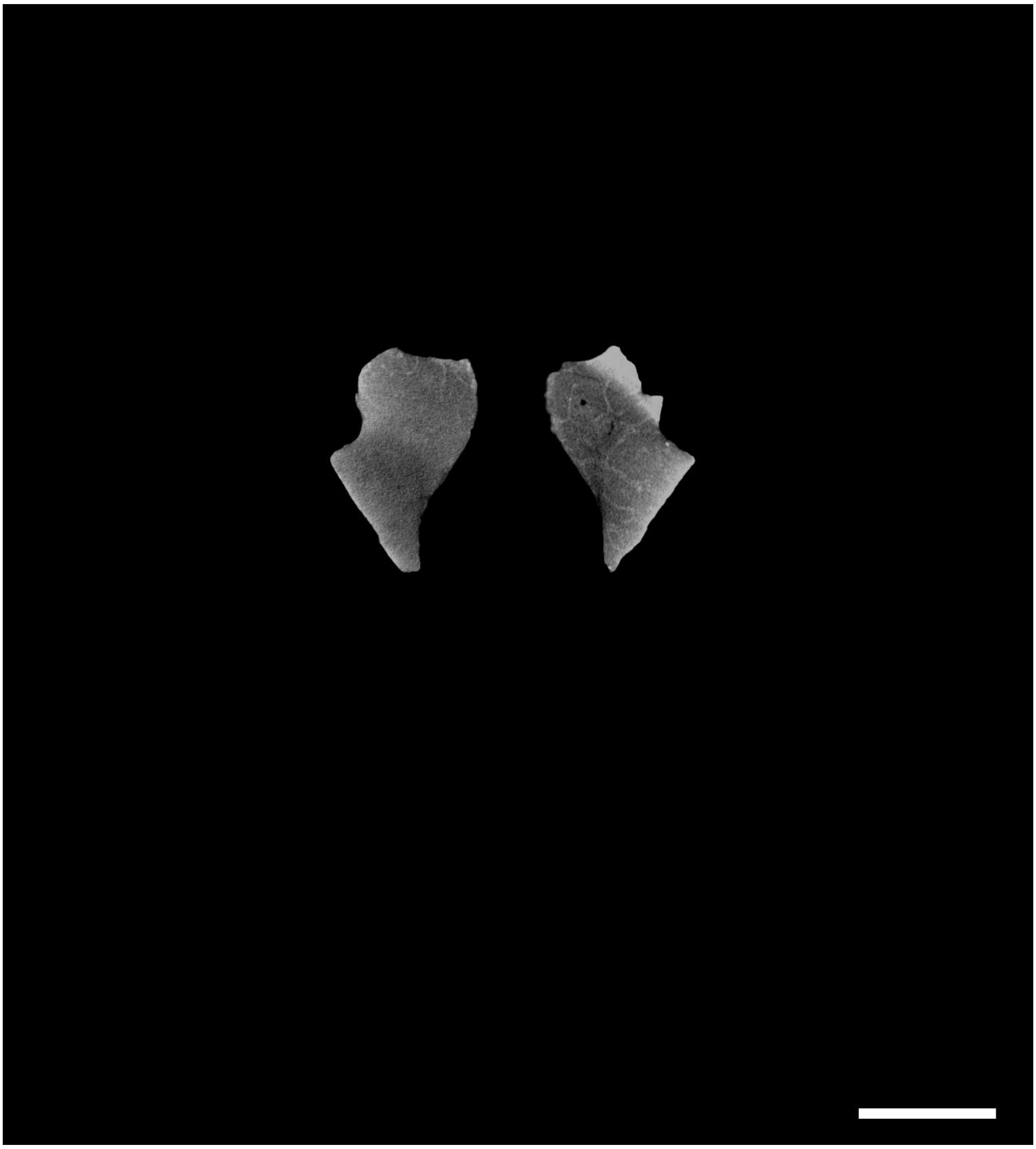

Supplementary Figure S14. Cross section of the postzygapophyses of the eighth cervical vertebra belongs to SNSB/BSPG 1991 I 27. Scale bar: 8 mm.

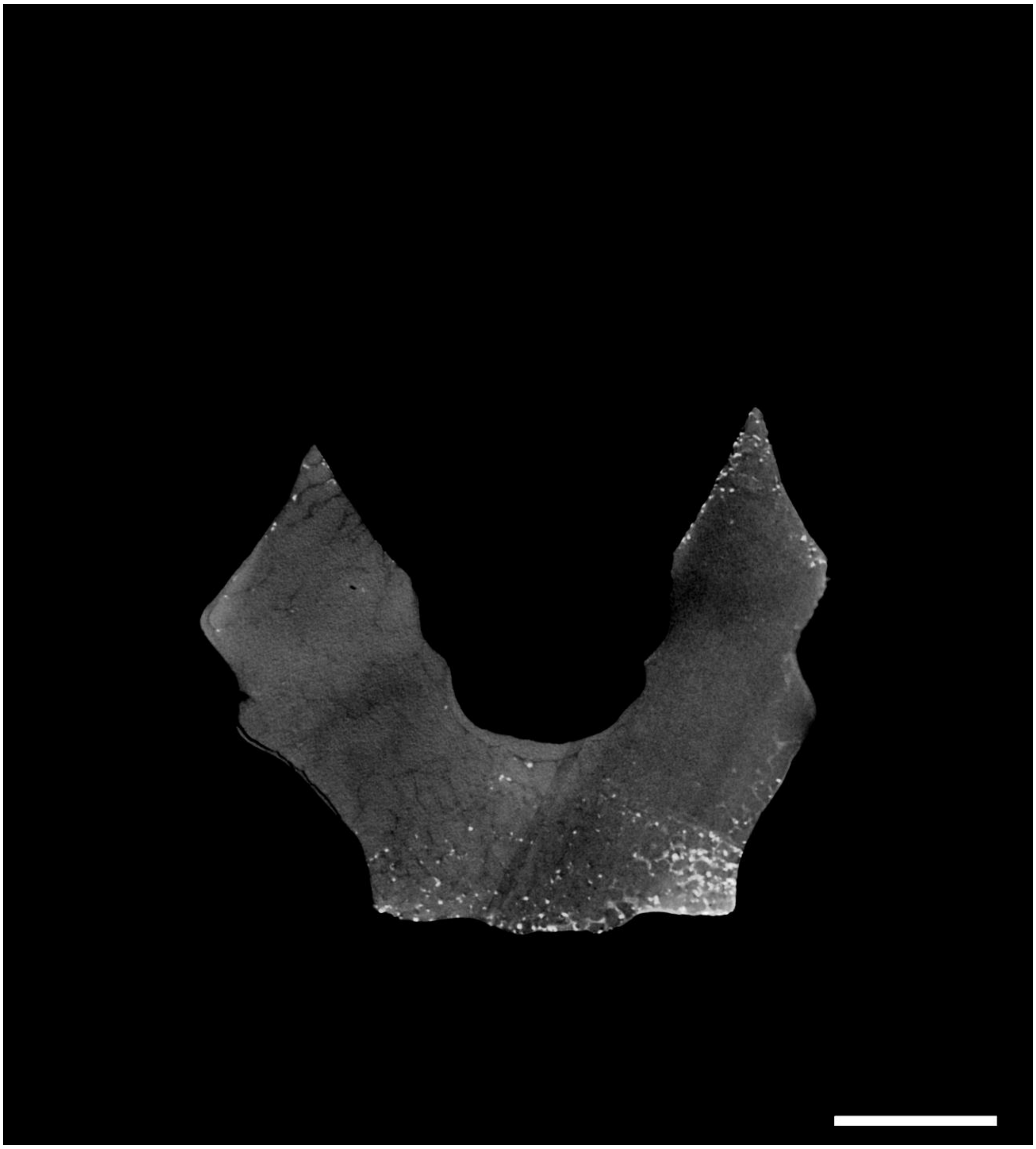

Supplementary Figure S15. Cross section of the cotyle of the ninth cervical vertebra belongs to SNSB/BSPG 1991 I 27. Scale bar: 8 mm.

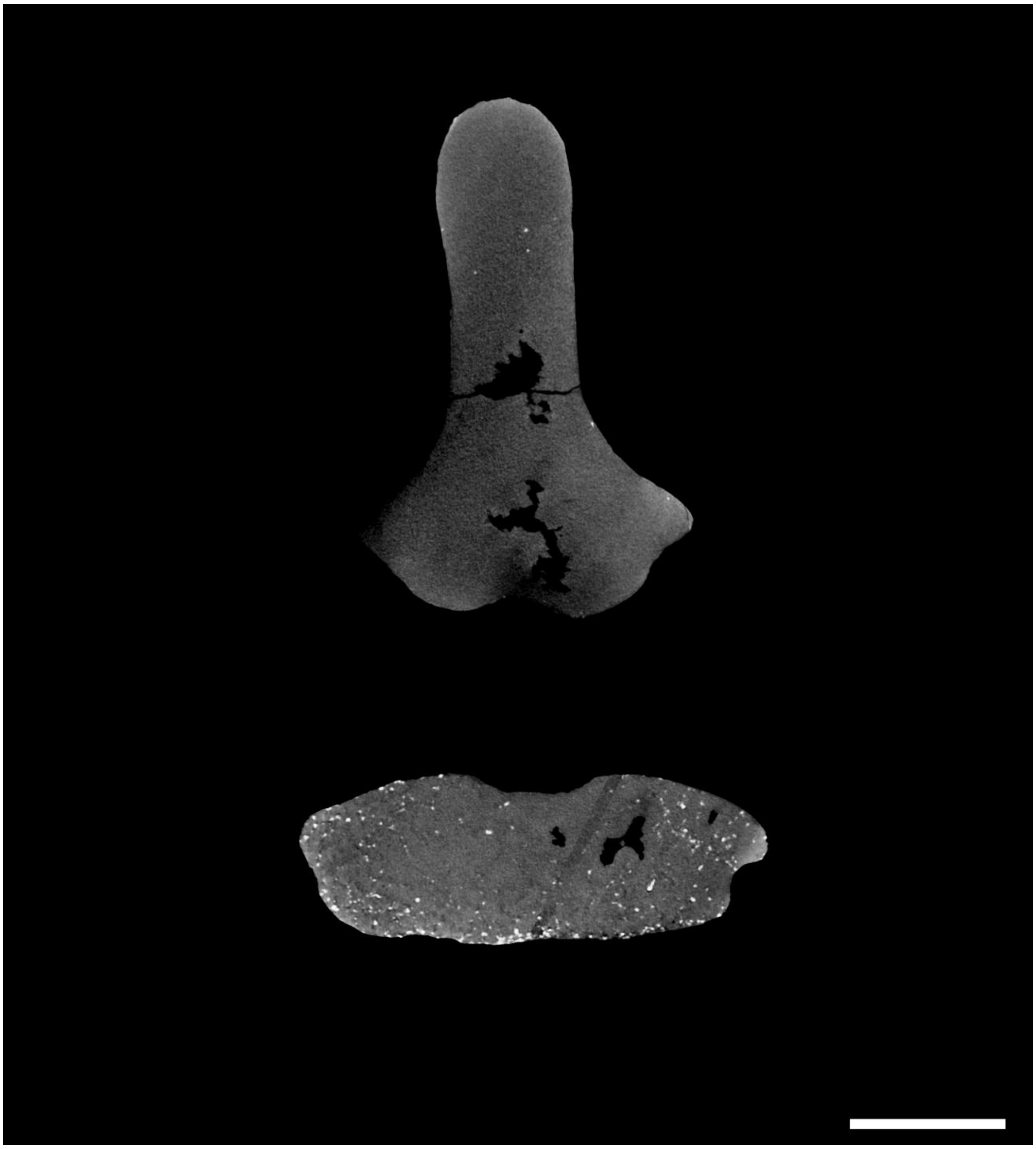

Supplementary Figure S16. Cross section of the condyle of the ninth cervical vertebra belongs to SNSB/BSPG 1991 I 27. Scale bar: 8 mm.

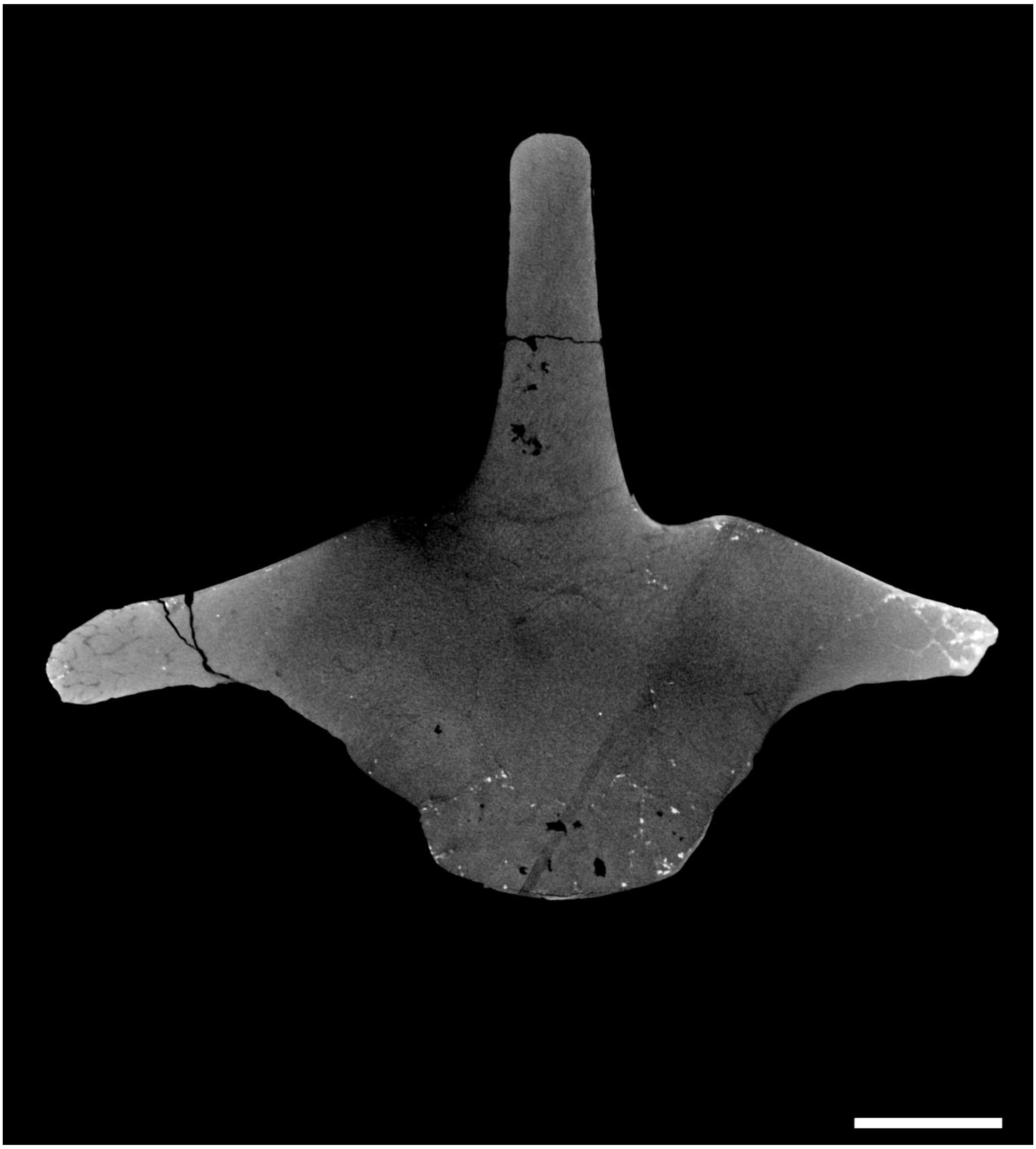

Supplementary Figure S17. Cross section of the mid-length of the ninth cervical vertebra belongs to SNSB/BSPG 1991 I 27. Scale bar: 8 mm.

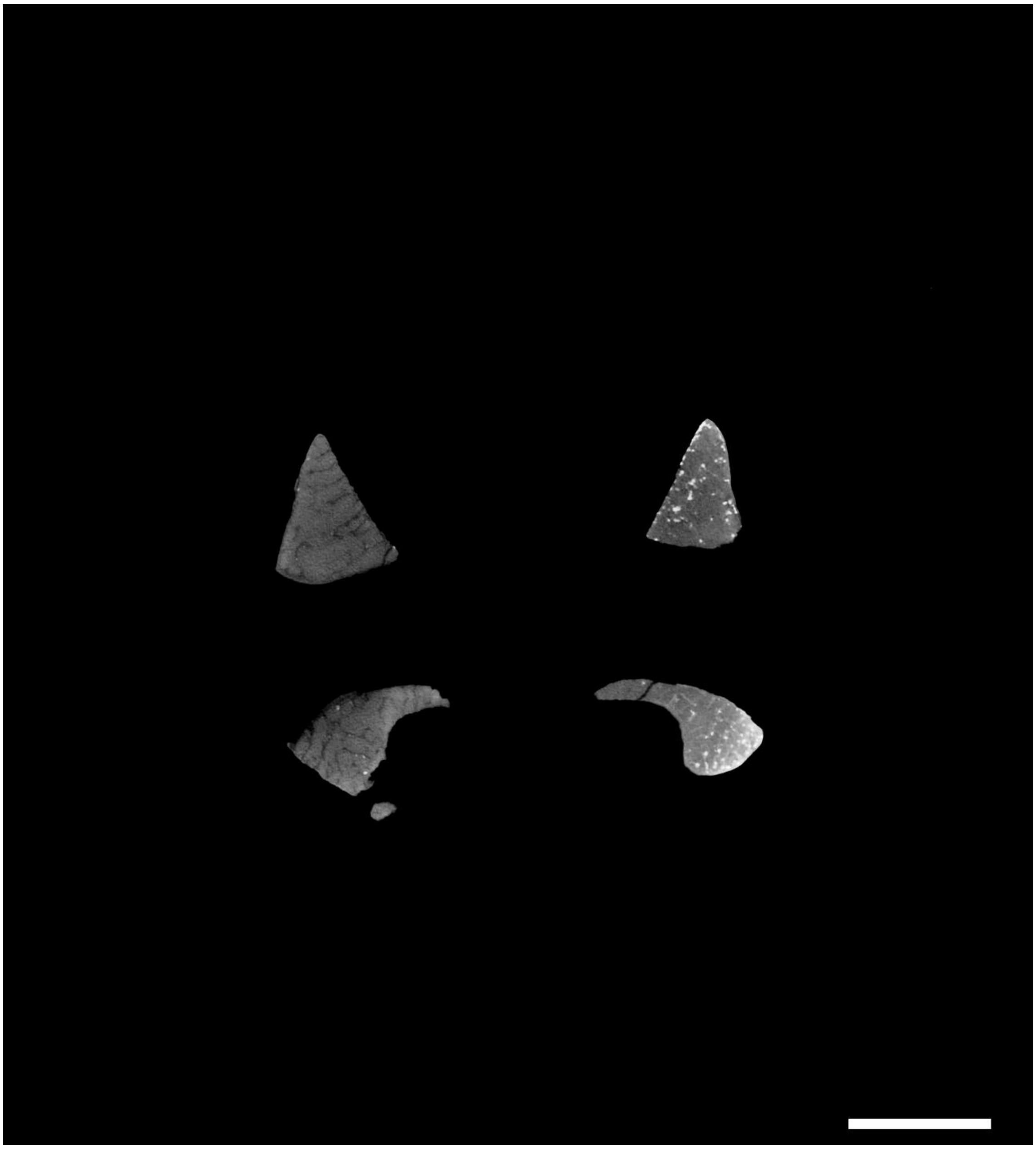

Supplementary Figure S18. Cross section of the prezygapophyses of the ninth cervical vertebra belongs to SNSB/BSPG 1991 I 27. Scale bar: 8 mm.

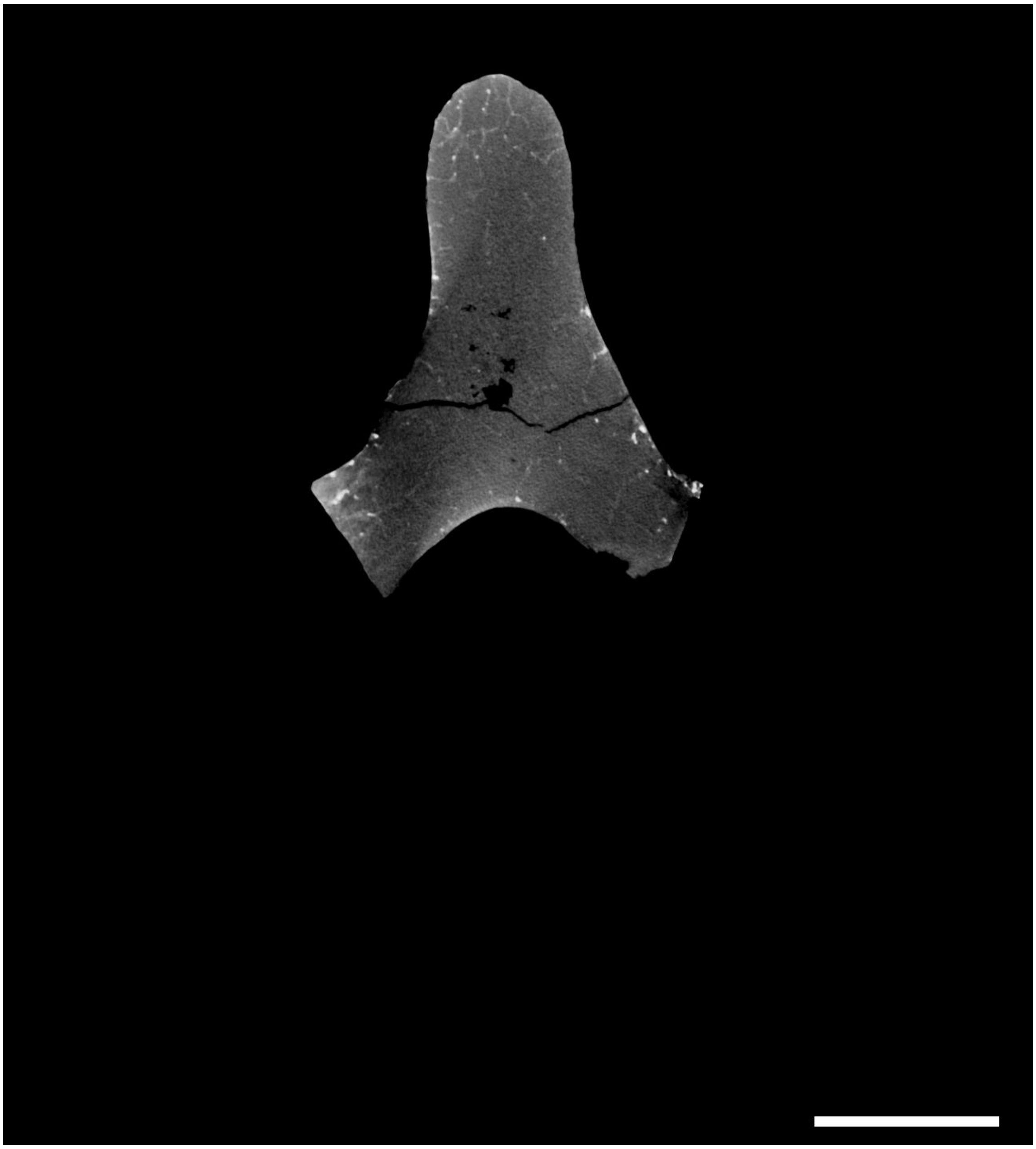

Supplementary Figure S19. Cross section of the postzygapophyses of the ninth cervical vertebra belongs to SNSB/BSPG 1991 I 27. Scale bar: 8 mm.

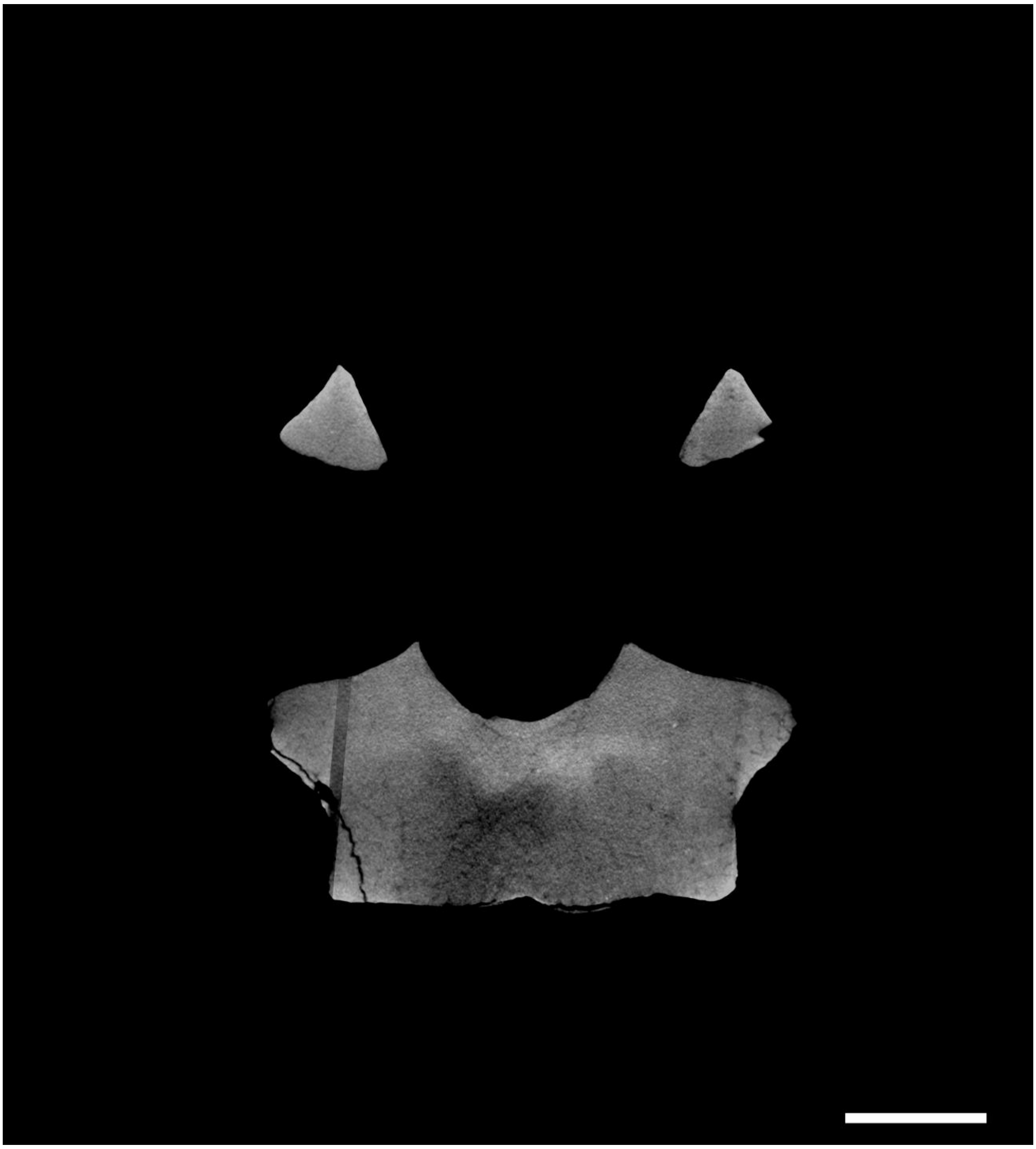

Supplementary Figure S20. Cross section of the cotyle of the first dorsal vertebra belongs to SNSB/BSPG 1991 I 27. Scale bar: 6.5 mm.

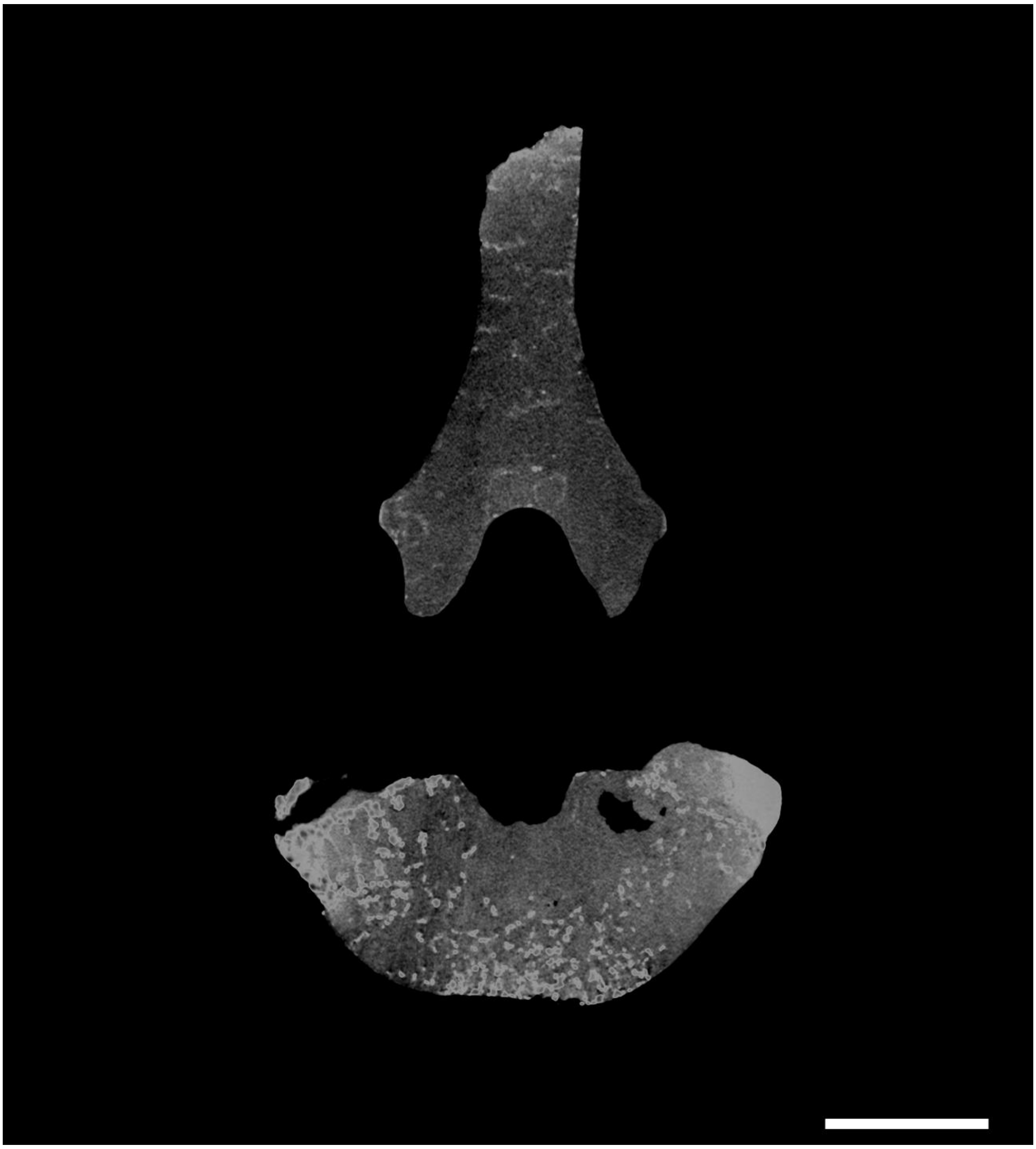

Supplementary Figure S21. Cross section of the condyle of the first dorsal vertebra belongs to SNSB/BSPG 1991 I 27. Scale bar: 6.5 mm.

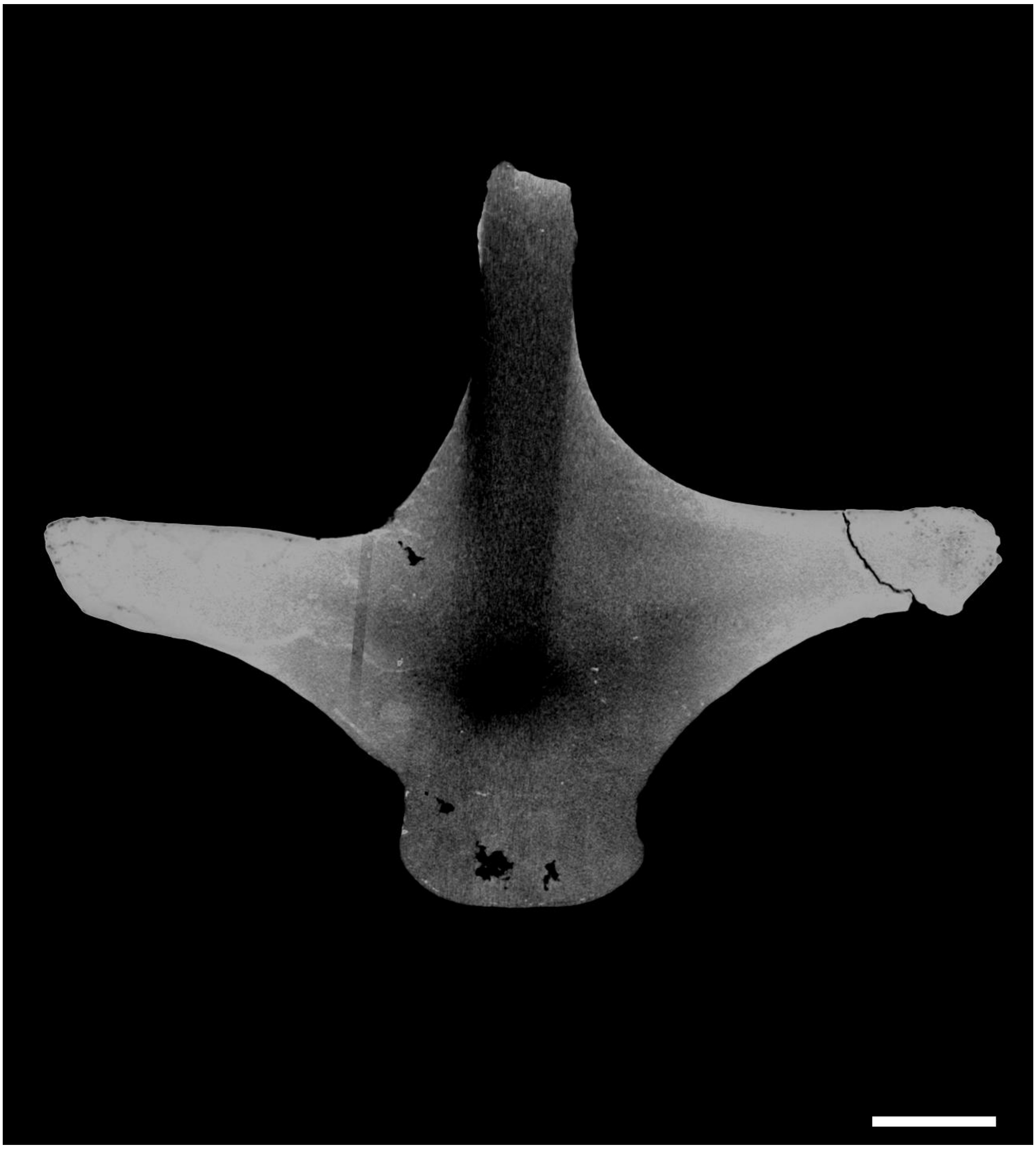

Supplementary Figure S22. Cross section of the mid-length of the first dorsal vertebra belongs to SNSB/BSPG 1991 I 27. Scale bar: 6.5 mm.

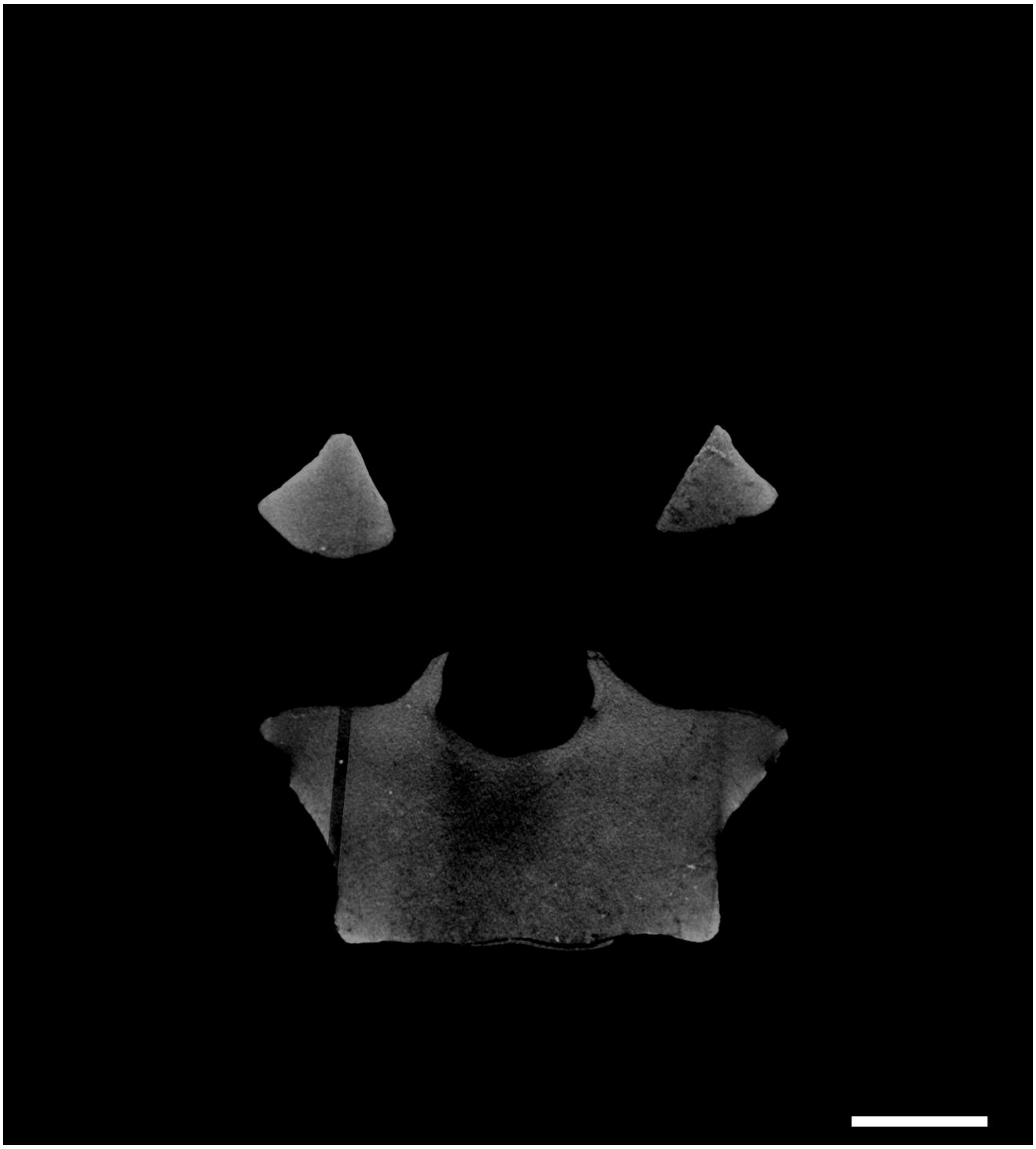

Supplementary Figure S23. Cross section of the prezygapophyses of the first dorsal vertebra belongs to SNSB/BSPG 1991 I 27. Scale bar: 6.5 mm.

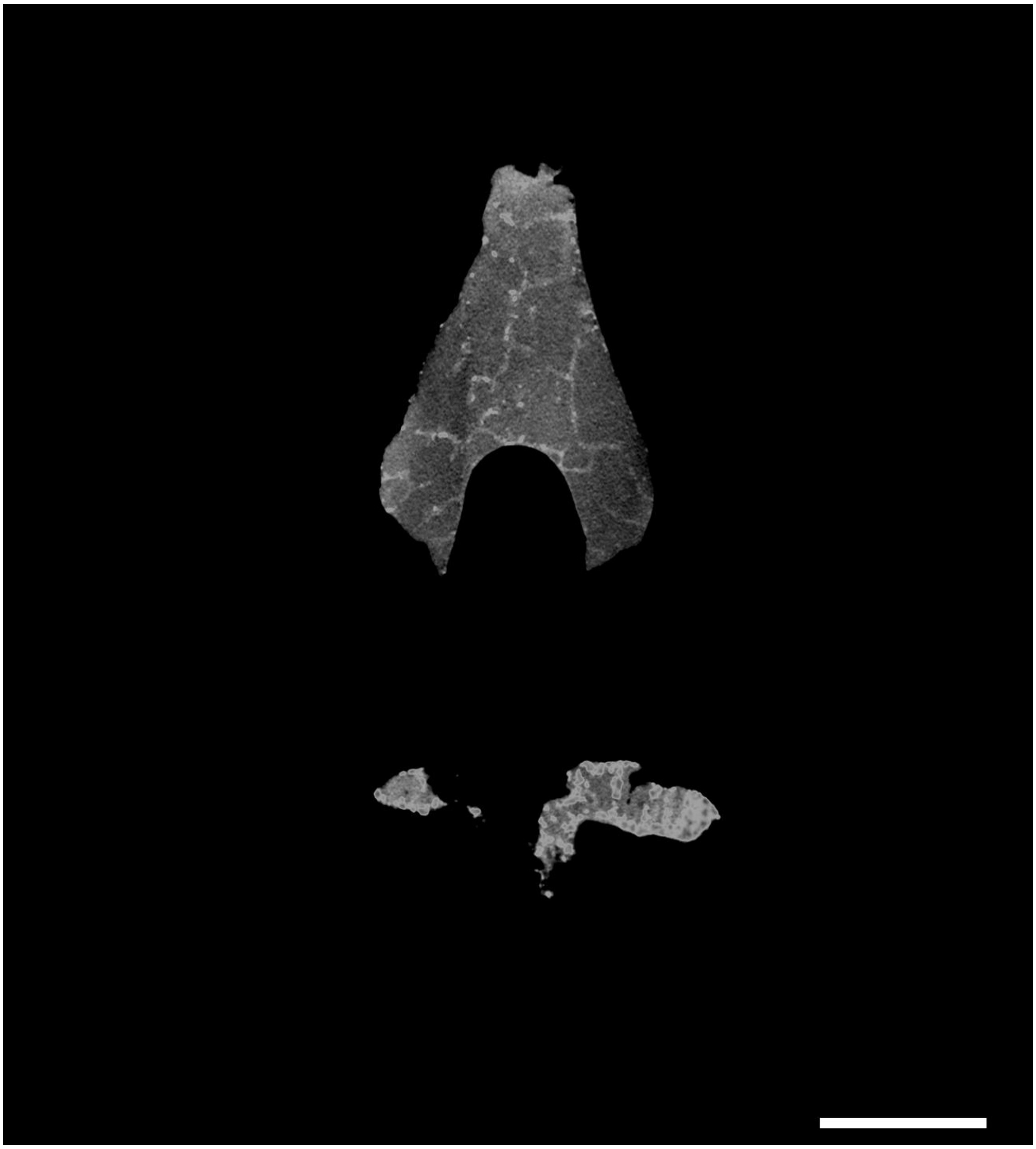

Supplementary Figure S24. Cross section of the postzygapophyses of the first dorsal vertebra belongs to SNSB/BSPG 1991 I 27. Scale bar: 6.5 mm.

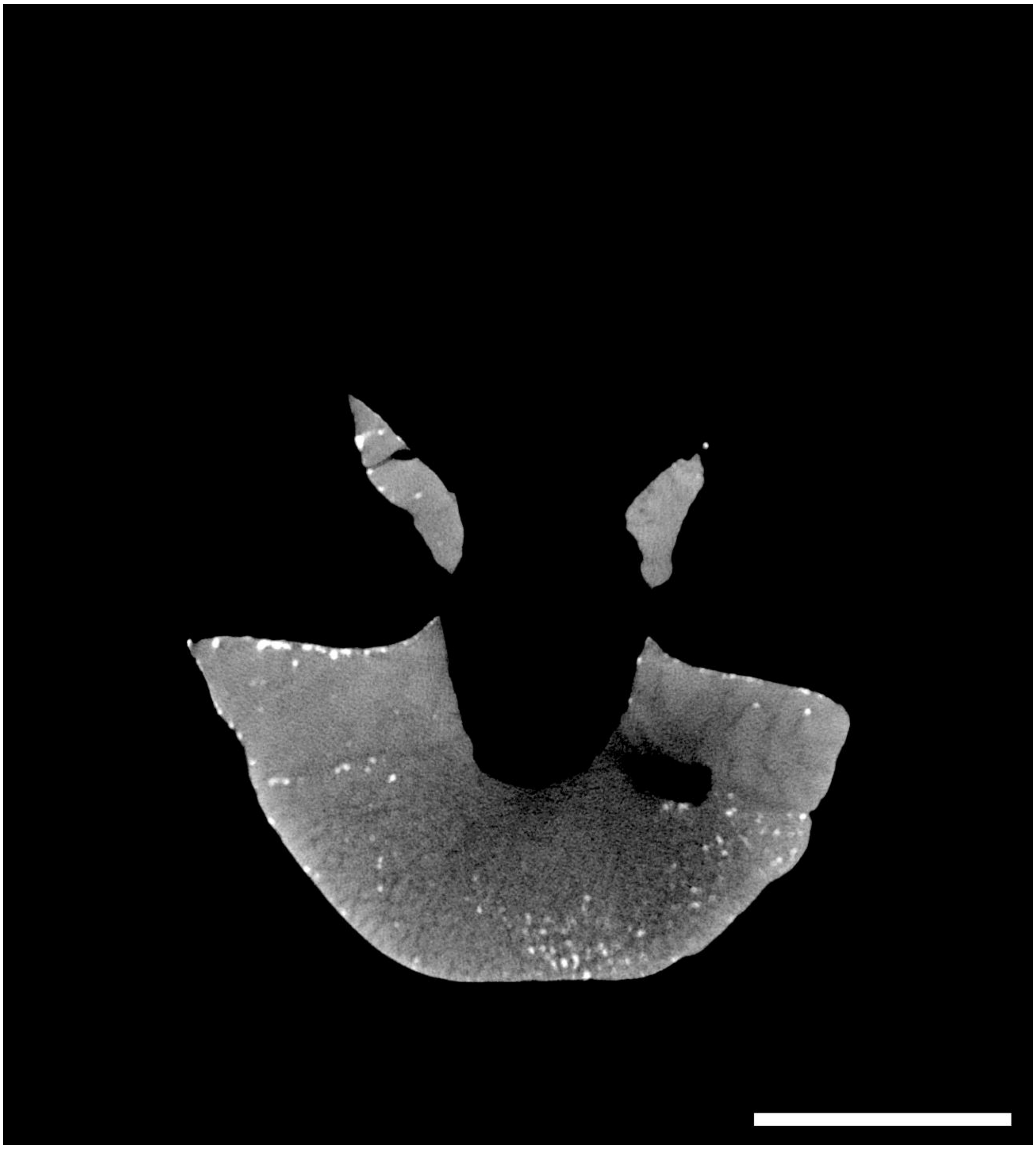

Supplementary Figure S25. Cross section of the cotyle and prezygapophyses of the second dorsal vertebra belongs to SNSB/BSPG 1991 I 27. Scale bar: 10 mm.

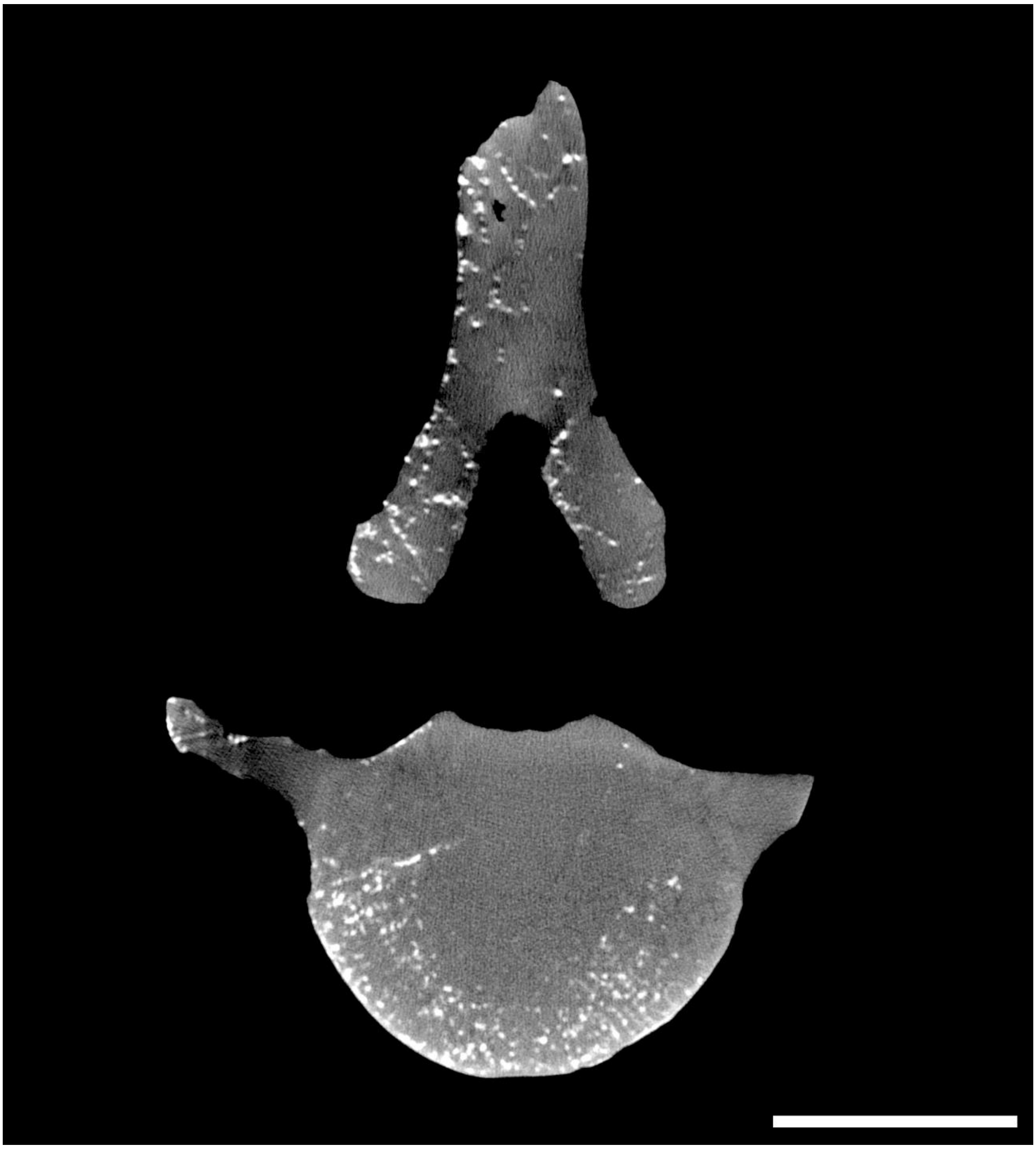

Supplementary Figure S26. Cross section of the condyle and postzygapophyses of the second dorsal vertebra belongs to SNSB/BSPG 1991 I 27. Scale bar: 10 mm.

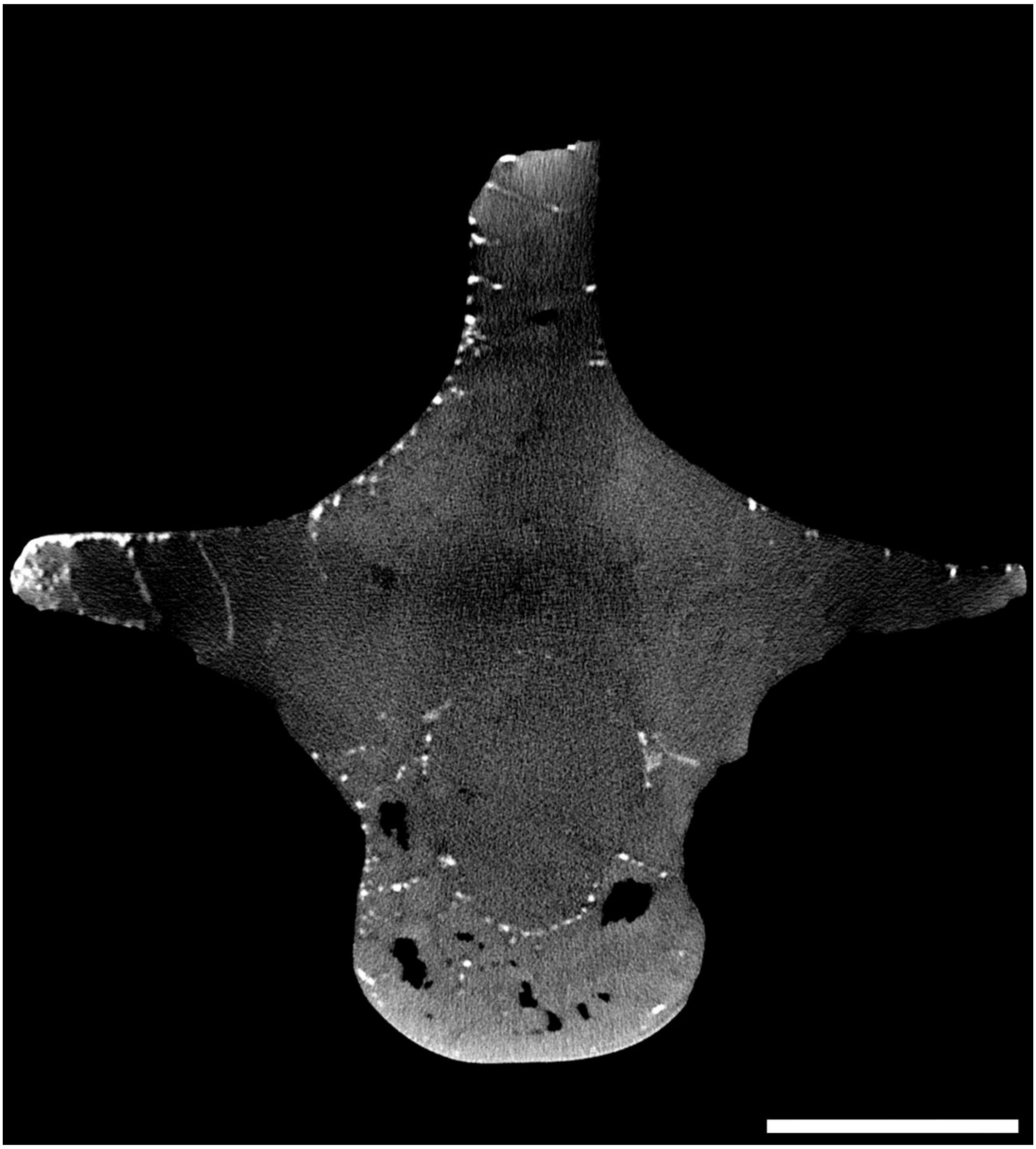

Supplementary Figure S27. Cross section of the mid-length of the second dorsal vertebra belongs to SNSB/BSPG 1991 I 27. Scale bar: 10 mm.

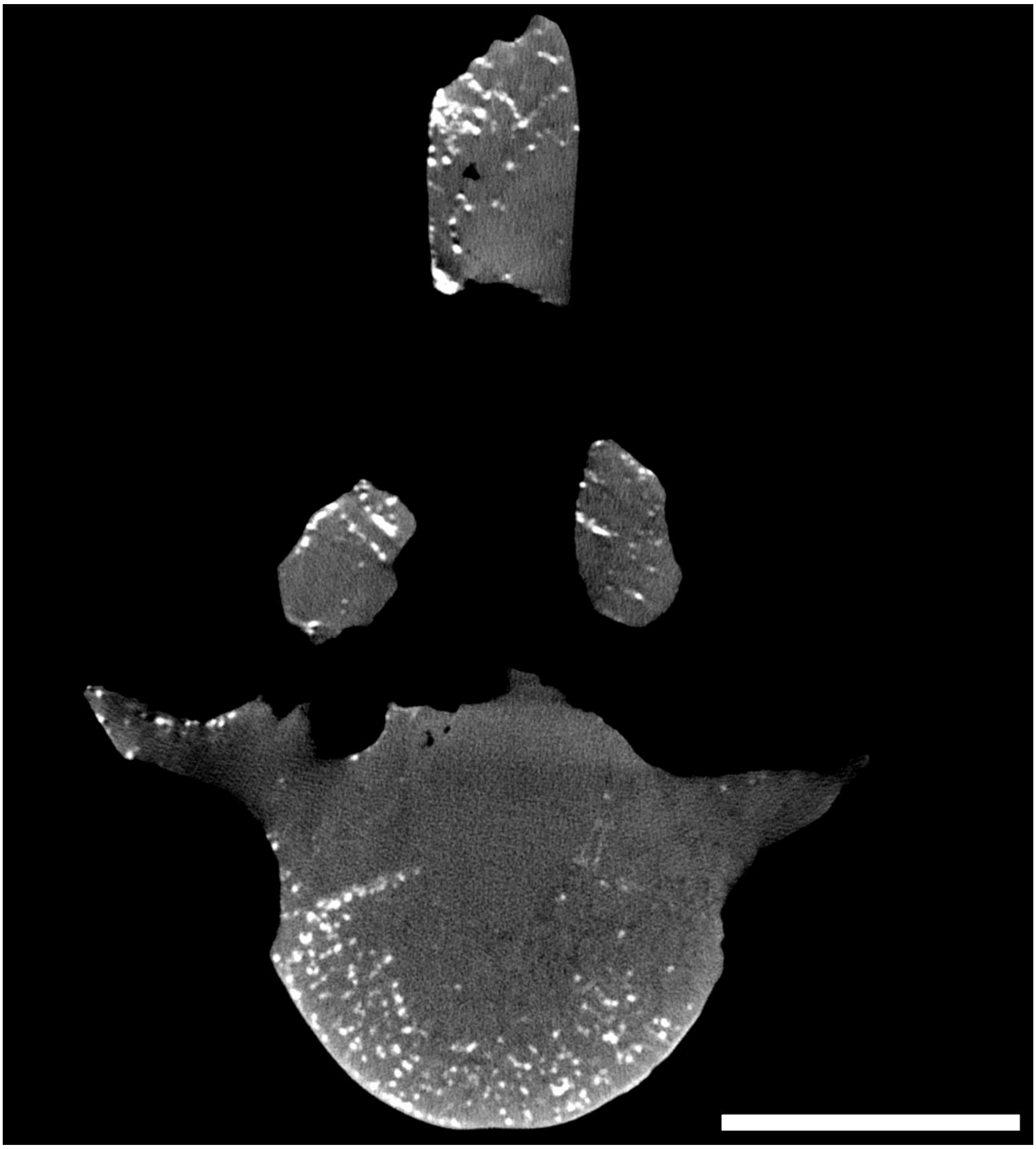

Supplementary Figure S28. Cross section of the cotyle and prezygapophyses of the third dorsal vertebra belongs to SNSB/BSPG 1991 I 27. Scale bar: 10 mm.

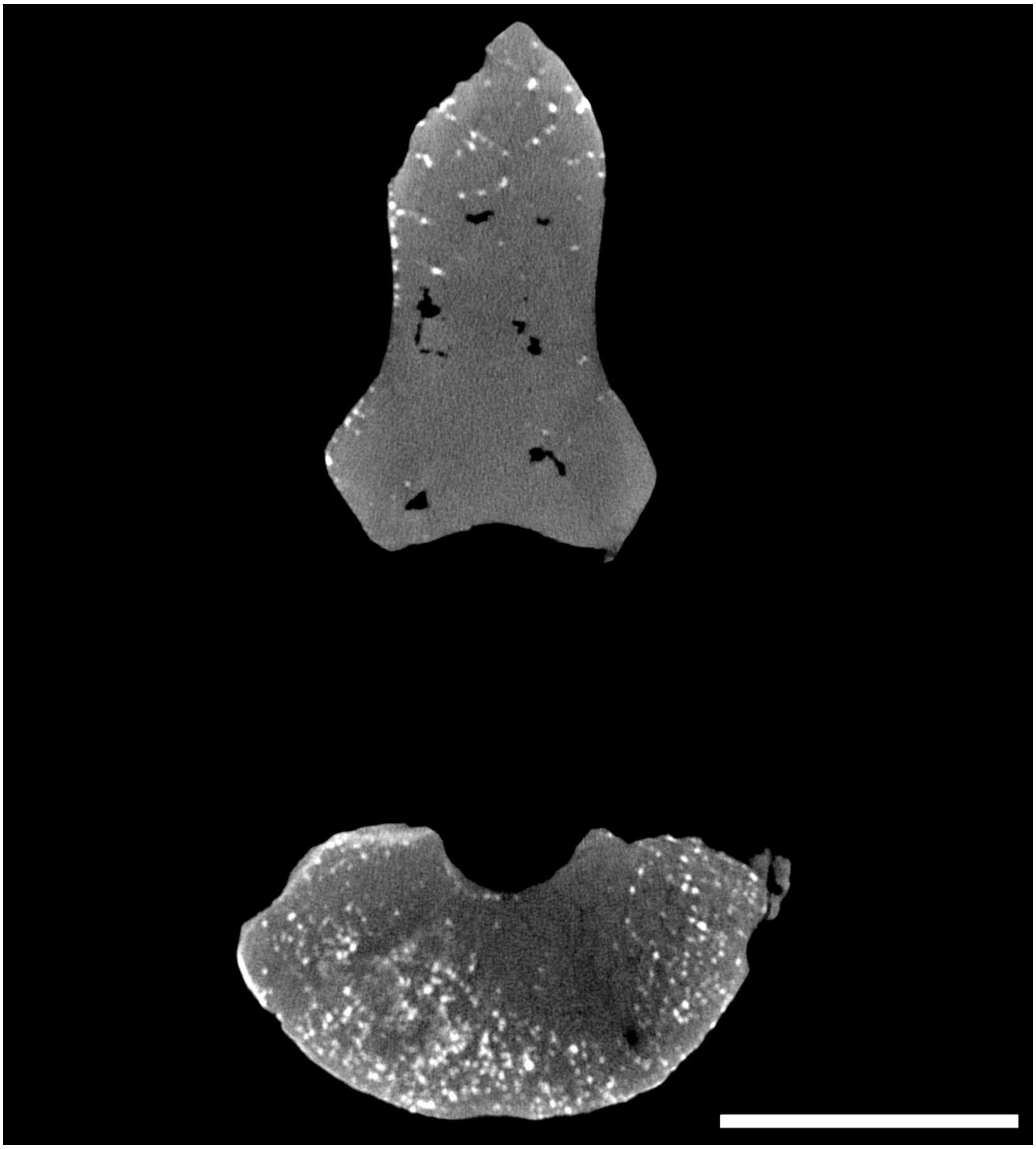

Supplementary Figure S29. Cross section of the condyle and postzygapophyses of the third dorsal vertebra belongs to SNSB/BSPG 1991 I 27. Scale bar: 10 mm.

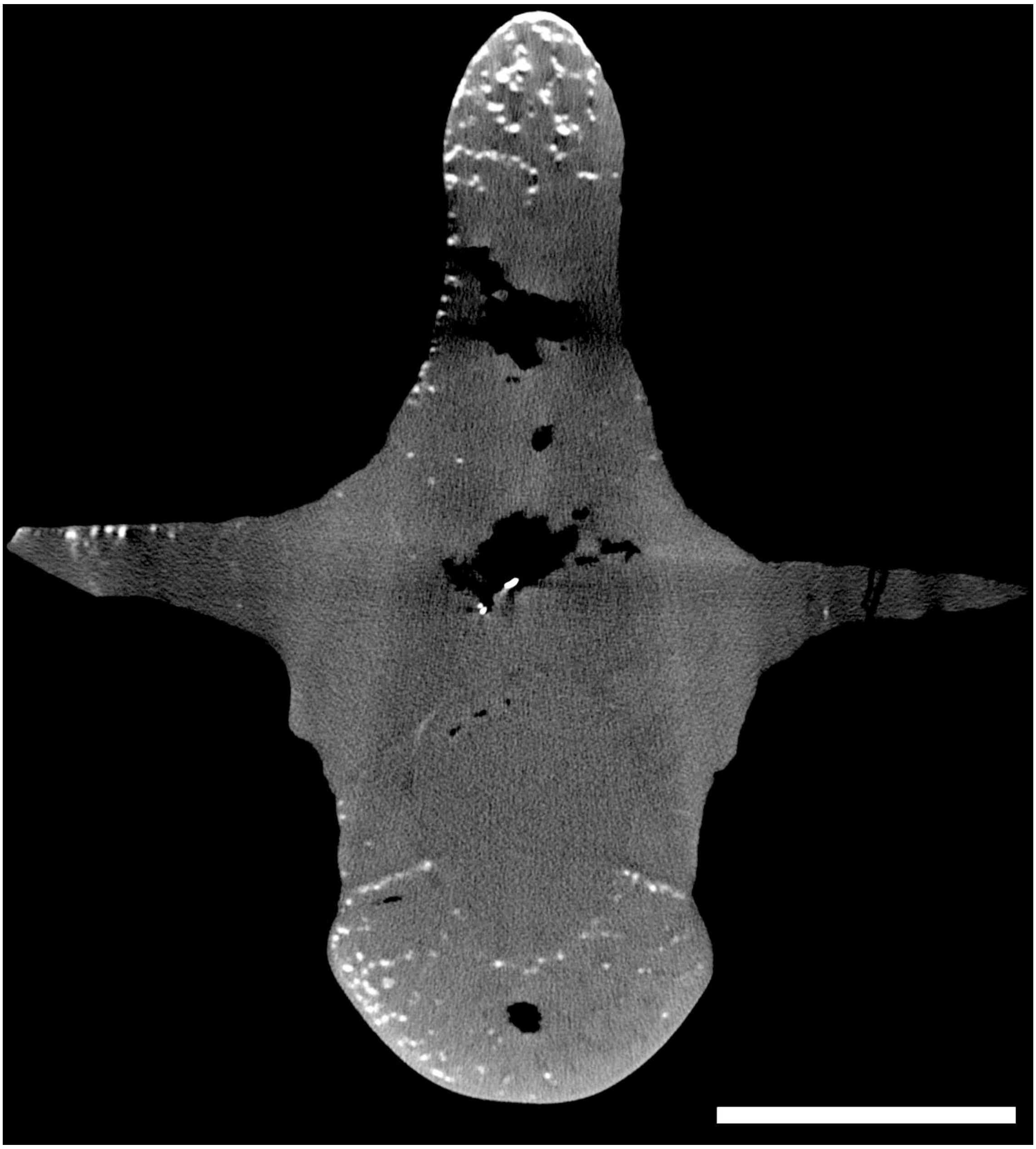

Supplementary Figure S30. Cross section of the mid-length of the third dorsal vertebra belongs to SNSB/BSPG 1991 I 27. Scale bar: 10 mm.

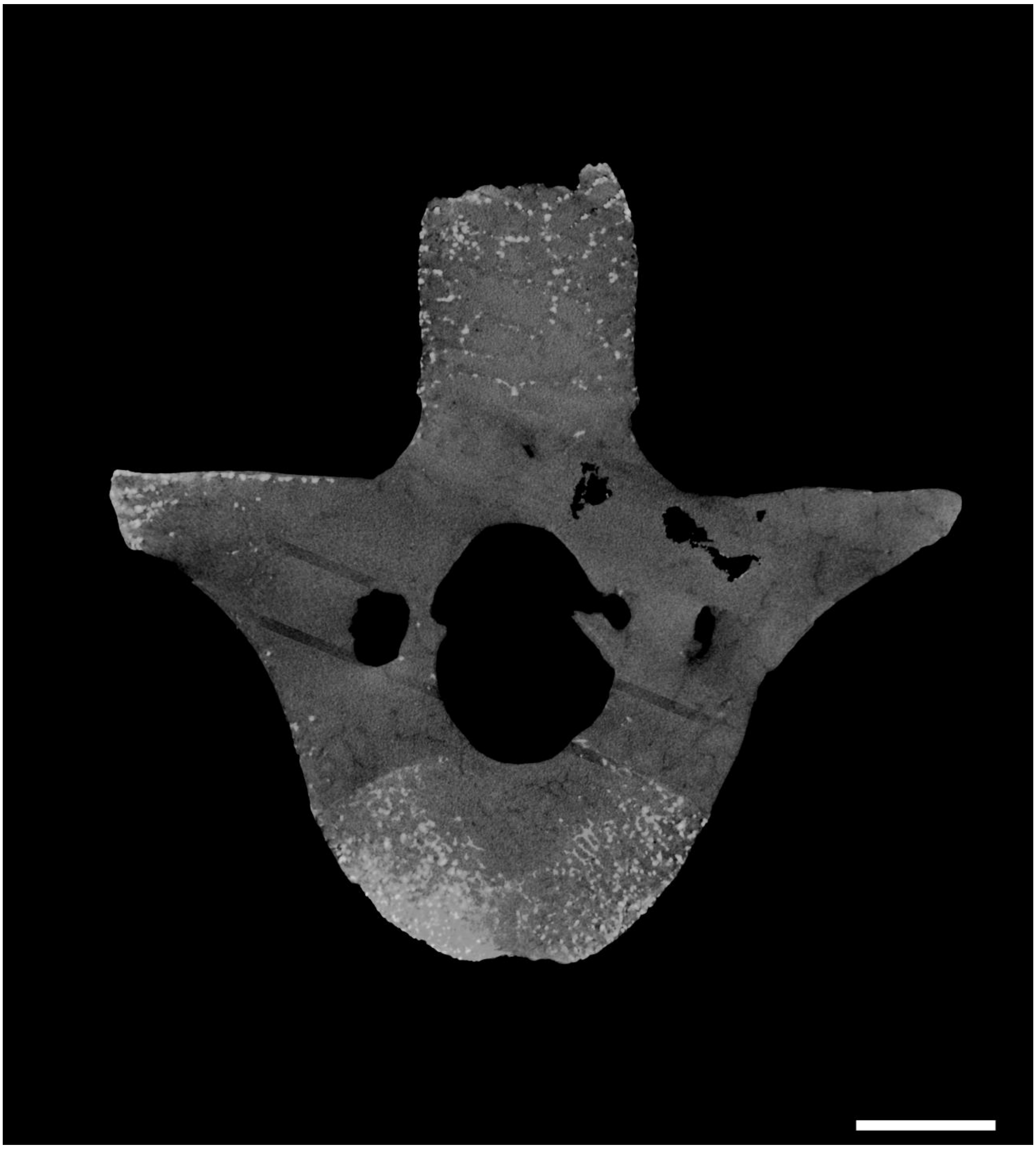

Supplementary Figure S31. Cross section of the cotyle of the fourth dorsal vertebra belongs to SNSB/BSPG 1991 I 27. Scale bar: 5.5 mm.

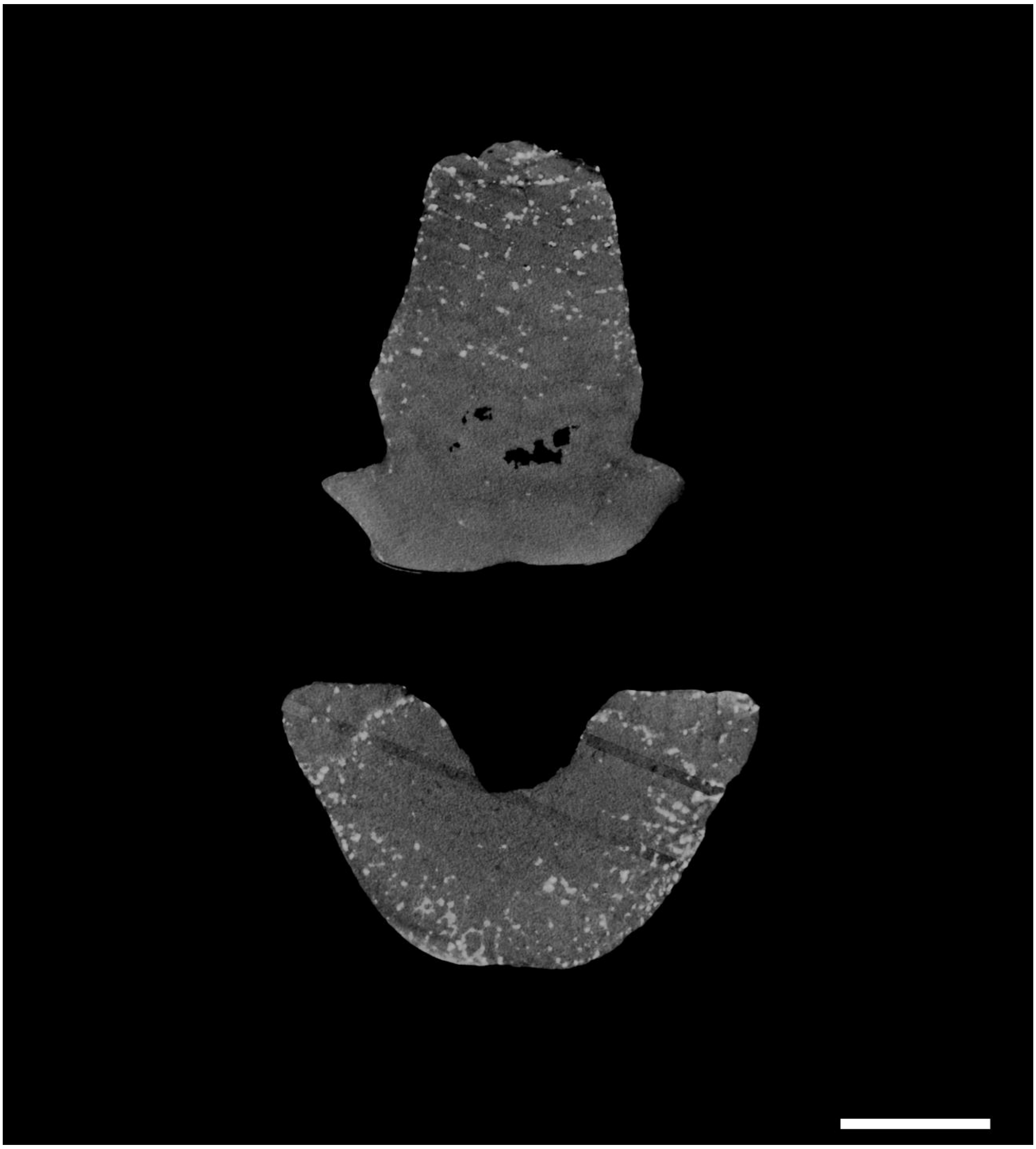

Supplementary Figure S32. Cross section of the condyle of the fourth dorsal vertebra belongs to SNSB/BSPG 1991 I 27. Scale bar: 5.5 mm.

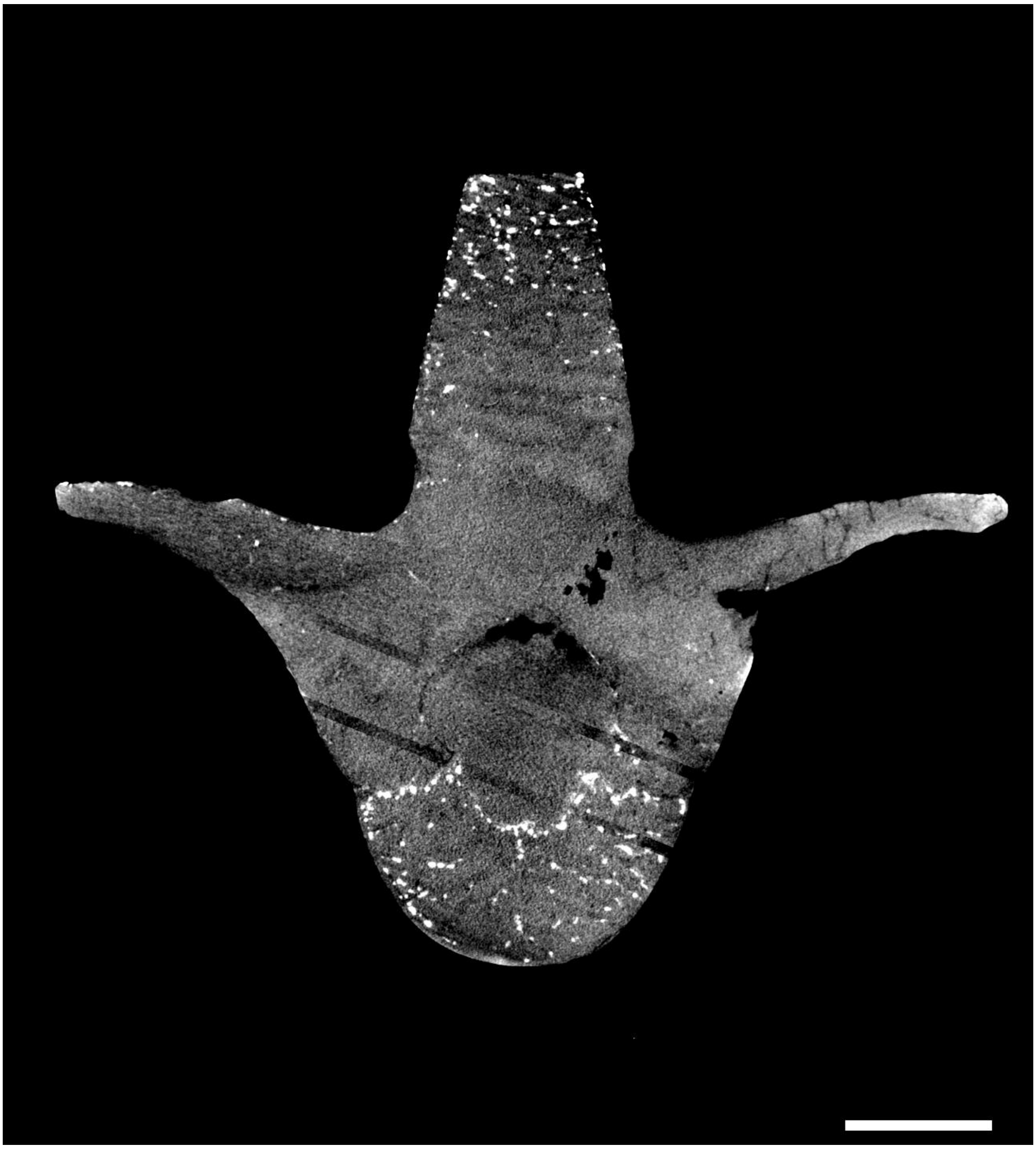

Supplementary Figure S33. Cross section of the mid-length of the fourth dorsal vertebra belongs to SNSB/BSPG 1991 I 27. Scale bar: 5.5 mm.

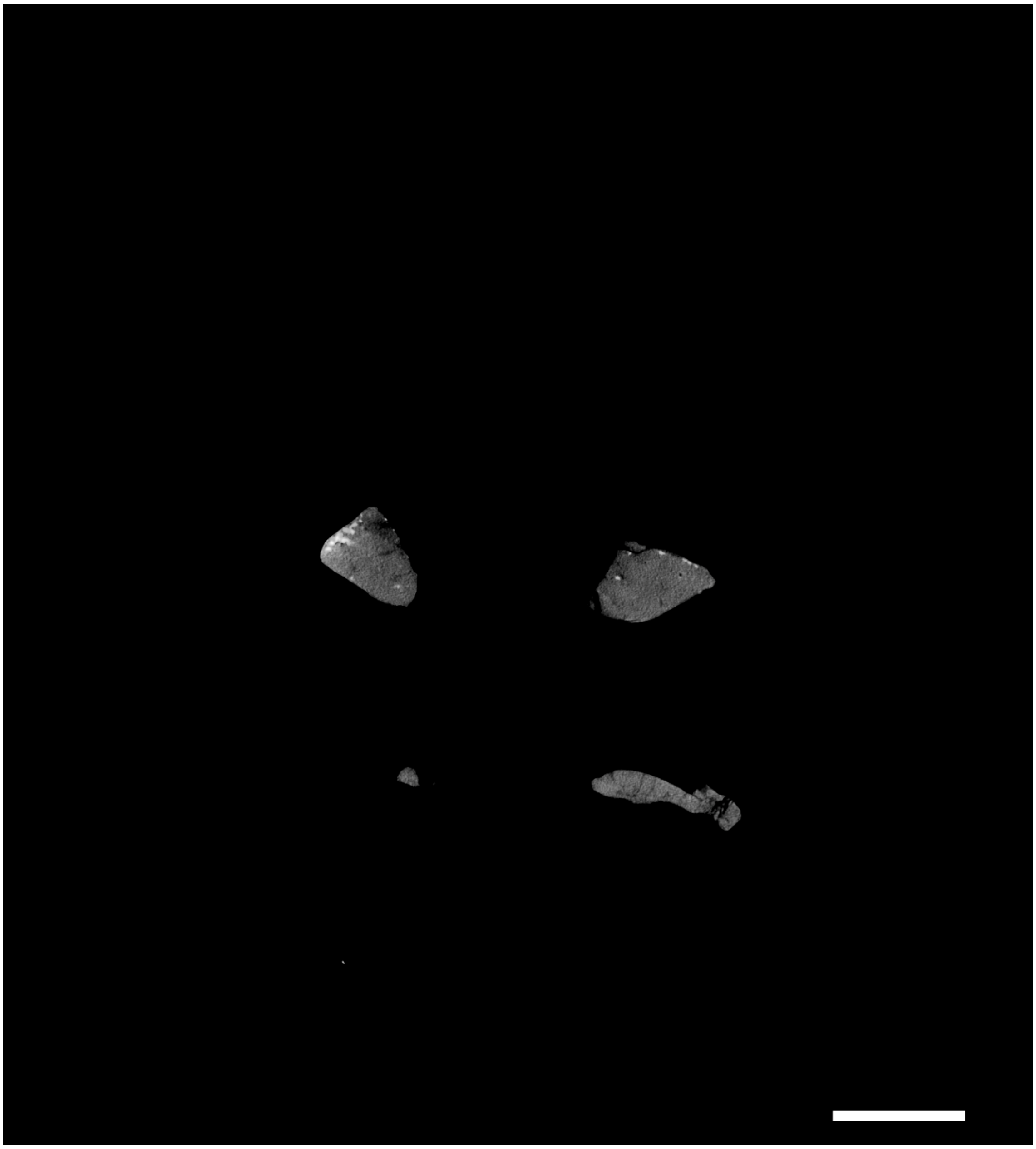

Supplementary Figure S34. Cross section of the prezygapophyses of the fourth dorsal vertebra belongs to SNSB/BSPG 1991 I 27. Scale bar: 5.5 mm.

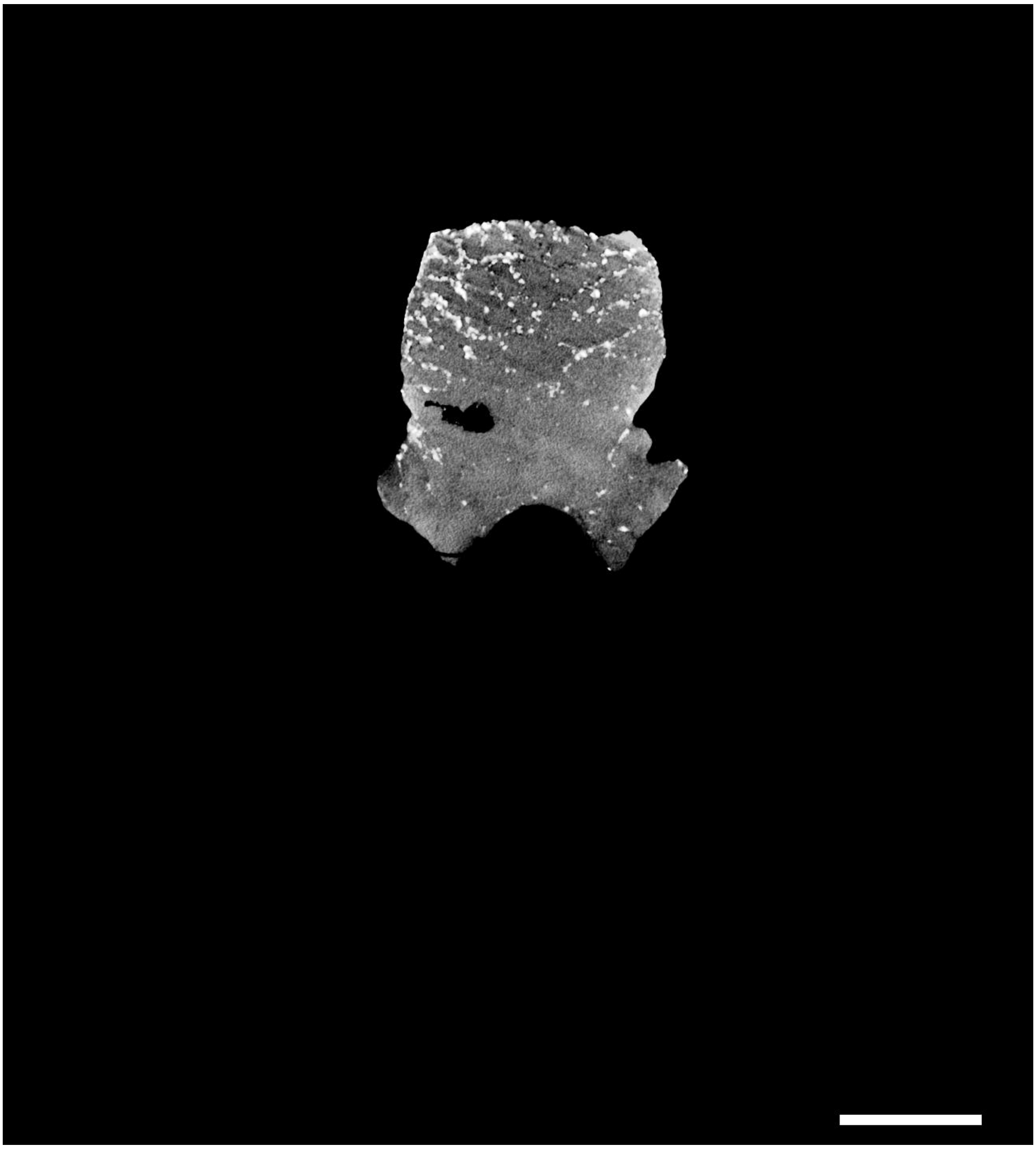

Supplementary Figure S35. Cross section of the postzygapophyses of the fourth dorsal vertebra belongs to SNSB/BSPG 1991 I 27. Scale bar: 5.5 mm.

Supplementary Table S1.
ASP values for each cross-section performed on all analyzed vertebrae

CV6 cross section	Air cavity area	Total area	ASP
Cotyle	866521	1186955	0.73
Centrum (mid-length)	2233504	3121806	0.71
Condyle	2908565	3854409	0.75
Left prezygapophysis	662470	973376	0.68
Right prezygapophysis	895997	1374804	0.65
Neural arch (mid-length)	4642904	5947205	0.78
Left postzygapophysis	1215437	1640075	0.74

CV7 cross section	Air cavity area	Total area	ASP
Cotyle	1019225	1433628	0.71
Centrum (mid-length)	2160616	2934246	0.73
Condyle	692523	1137992	0.60
Right prezygapophysis	593136	849499	0.69
Neural arch (mid-length)	3427144	4325759	0.79

CV8 cross section	Air cavity area	Total area	ASP
Cotyle	483282	687322	0.70
Centrum (mid-length)	327434	471085	0.69
Condyle	209183	361570	0.57
Left prezygapophysis	168919	221769	0.76
Right prezygapophysis	186790	252370	0.74
Neural arch (mid-length)	1861685	2312030	0.80
Left postzygapophysis	261631	323170	0.80
Right postzygapophysis	210096	285939	0.73

CV9 cross section	Air cavity area	Total area	ASP
Cotyle	270826	483257	0.56
Centrum (mid-length)	282473	382295	0.73
Condyle	598667	854663	0.70
Left prezygapophysis	129554	161859	0.80
Right prezygapophysis	73566	116793	0.62
Neural arch (mid-length)	2374731	2804255	0.84
Left postzygapophysis	76144	110211	0.69
Right postzygapophysis	67327	93469	0.72

**DV1 cross section**	**Air cavity area**	**Total area**	**ASP**
Cotyle	381855	588933	0.64
Centrum (mid-length)	501037	680380	0.73
Condyle	573014	954490	0.60
Left prezygapophysis	81219	120070	0.67
Right prezygapophysis	132918	181018	0.73
Neural arch (mid-length)	3326852	4080773	0.81
Left postzygapophysis	66013	97974	0.67
Right postzygapophysis	62941	100045	0.62

**DV2 cross section**	**Air cavity area**	**Total area**	**ASP**
Cotyle	3082016	4379428	0.70
Centrum (mid-length)	1114304	1760239	0.63
Condyle	990144	1709206	0.57
Left prezygapophysis	123624	178938	0.69
Right prezygapophysis	192894	255137	0.75
Neural arch (mid-length)	6327006	8117911	0.77
Left postzygapophysis	249341	373510	0.66
Right postzygapophysis	228364	327534	0.69

**DV3 cross section**	**Air cavity area**	**Total area**	**ASP**
Cotyle	2232654	3323451	0.67
Centrum (mid-length)	1485526	2308465	0.64
Condyle	3087266	4524029	0.68
Left prezygapophysis	396701	568996	0.69
Right prezygapophysis	368653	564975	0.65
Neural arch (mid-length)	8443265	10233545	0.82
Left postzygapophysis	304179	402527	0.75
Right postzygapophysis	323750	407303	0.79

**DV4 cross section**	**Air cavity area**	**Total area**	**ASP**
Cotyle	520633	915405	0.56
Centrum (mid-length)	443436	684363	0.64
Condyle	609991	1140030	0.53
Left prezygapophysis	81387	110998	0.73
Right prezygapophysis	90139	119240	0.75
Neural arch (mid-length)	2303612	2804313	0.82
Left postzygapophysis	96946	134178	0.72
Right postzygapophysis	76188	112253	0.67
